# Sex differences in primary Sjögren’s disease: prognostic impact on mortality and cancer

**DOI:** 10.1186/s13293-026-00827-7

**Published:** 2026-01-14

**Authors:** Chunxin Lei, Xiya Zhang, Yan Zhang, Yunlong Fang, Jiaqi Chen, Zihan Liu, Xuanyi Zhou, Bojie Tang, Jiahe Liao, Ziwei Huang, Jianying Yang, Zihan Wang, Ting Liu, Qingwen Tao, Jing Luo

**Affiliations:** 1 Graduate School, Beijing University of Chinese Medicine, Beijing, China; 2 National Center for Integrative Medicine, Department of TCM Rheumatism, China-Japan Friendship Hospital, Beijing, People’s Republic of China

**Keywords:** Sjögren’s disease, Prognostic factor, Male sex, Mortality, Cancer

## Abstract

**Objective:**

To assess the impact of sex on mortality and malignancy in patients with primary Sjögren’s disease (pSD).

**Methods:**

This ambispective cohort study included 1,182 pSD patients (1,025 women and 157 men) from the China–Japan Friendship Hospital between 2014–2023 and followed through 2024. Survival outcomes were estimated via Kaplan–Meier curves, and SMRs were calculated. Independent risk factors were identified via multivariate Cox regression, followed by stepwise Cox, sex-stratified and interaction analyses.

**Results:**

During follow-up, 125 deaths (10.6%) and 33 malignancies (2.8%) occurred, both of which were more common in men (log–rank tests, *P* < 0.001). Overall mortality was markedly higher in men (25.5%, SMR = 4.79) than in women (8.3%, SMR = 2.42). Male sex was independently associated with higher risk of mortality (HR = 1.998, *P* = 0.004) and cancer incidence (HR = 3.799, *P* = 0.001). Sex-stratified analyses revealed distinct death–associated factors: interstitial lung disease (ILD), pulmonary infection and ischemic stroke were independently associated with mortality in men, whereas older age, low C3, elevated C-reactive protein and total bilirubin (TBIL) levels were associated with mortality in women. With respect to cancer, longer disease duration, lymphadenopathy, lymphocytopenia, elevated TBIL and hypochloremia were independently associated with cancer risk in women, whereas no variables showed an independent association with cancer risk among men. Interaction analyses demonstrated additive interactions between male sex and elevated ESSDAI scores, as well as male sex and concomitant ILD, in relation to mortality.

**Conclusion:**

Compared with women, men with pSD presented greater risks and distinct risk profiles of death and malignancy. Moreover, male sex was independently associated with adverse outcomes and acted as an important effect modifier, highlighting the need for sex-specific risk stratification and clinical management.

**Trial registration:**

This ambispective cohort study was registered in the Clinical Trial Registry (ID: NCT06528197) on June 27, 2024 and conducted in accordance with the Declaration of Helsinki.

**Supplementary Information:**

The online version contains supplementary material available at 10.1186/s13293-026-00827-7.

## Abstract (plain language summary)

Primary Sjögren’s disease (pSD) is an autoimmune disorder that causes dryness of the eyes and mouth, fatigue, and damage to multiple organs. While pSD mainly affects women, men with the disease tend to experience more severe complications. We enrolled 1,182 patients in China (1,025 women and 157 men) and compared disease features, mortality, and cancer outcomes between male and female patients. During follow-up, about 25% of the men died, compared to about 8% of the women. Sex-stratified analyses revealed distinct prognostic profiles, such as ILD, infections, and ischemic stroke, were particularly dangerous for men, whereas age, inflammation, and low complement levels associated with poor outcomes in women. Male sex remained independently associated with increased risk even after accounting for these factors. These results show that men with pSD represent a distinct, high-risk subgroup who require closer monitoring and tailored treatment strategies. Underscoring the need for sex-specific risk stratification, vigilant monitoring, and tailored management.

**Supplementary Information:**

The online version contains supplementary material available at 10.1186/s13293-026-00827-7.

## Highlights

- Male patients with primary Sjögren’s disease (pSD) exhibited markedly higher mortality (25.5%) and cancer incidence (6.4%) than females (8.3% and 2.2%, respectively).
- Male sex was independently associated with a higher risk of mortality (HR = 1.998) and cancer incidence (HR = 3.799) after multivariable adjustment, confirming its robust prognostic value.
- Sex-stratified analyses revealed distinct prognostic profiles: ILD, infection, and ischemic stroke were associated with death in men, whereas older age, low C3, elevated CRP and TBIL were associated with death in women.
- Interaction analysis demonstrated significant additive interactions between male sex and high ESSDAI scores or concomitant ILD in relation to death. Our findings establish male sex is independently associated with adverse outcomes and acts as a significant effect modifier in pSD, underscoring the need for sex-specific risk stratification, vigilant monitoring, and tailored management.

**Supplementary Information:**

The online version contains supplementary material available at 10.1186/s13293-026-00827-7.

## Introduction

Primary Sjögren’s disease (pSD) is a chronic systemic autoimmune disorder characterized by lymphocytic infiltration of exocrine glands—particularly the salivary and lacrimal glands—leading to xerostomia and xerophthalmia [1]. In addition to glandular dysfunction, pSD frequently involves multiple organ systems, manifesting as interstitial lung disease (ILD), renal tubular acidosis, and peripheral neuropathy. Patients also exhibit an elevated risk of developing lymphomas [2]. The estimated incidence of pSD ranges from 1–10 cases per 100,000 person-years [3]. The disease predominantly affects women [4, 5], with reported female-to-male incidence ratios between 9:1 and 22.9:1 in recent epidemiological studies [6–8]. This striking sex disparity corresponds with distinct phenotypic profiles [9, 10]. Women with pSD typically present with prominent glandular manifestations, whereas men—although less frequently affected—tend to develop more severe extraglandular disease and experience adverse outcomes, including increased risks of lymphoma and mortality [9, 10]. These differences likely reflect a complex interplay among sex hormones, X chromosome–linked mechanisms, reproductive factors, and environmental influences [11, 12]. Similar sex-based disparities are well documented in systemic lupus erythematosus (SLE), where men, despite having a lower disease prevalence, exhibit more severe extraglandular involvement, faster accrual of irreversible organ damage, and lower survival rates than women do [13–15]. Whether such sex-specific prognostic patterns extend to pSD remains incompletely understood.

Two meta-analyses incorporating multicenter data have shown that overall mortality in pSD patients exceeds that in the general population, with pooled standardized mortality ratios (SMRs) ranging from 1.38–1.46 [16, 17]. Several studies have further identified extraglandular involvement—particularly vasculitis, ILD, and cryoglobulinemia—as major predictors of death and lymphoma development [3, 18–20]. In Asia, SMRs have been reported from cohorts in Korea, Turkey, and Taiwan, while recent data from mainland China also confirmed increased mortality in pSD patients and identified male sex as a potential independent risk factor [21–25]. However, the influence of sex on mortality remains insufficiently defined. Large-scale cohort studies with adequate statistical power to explore sex-related variations in cancer and survival outcomes are scarce. Moreover, the potential interactive effects between sex and other prognostic variables have not been formally evaluated. No prior studies have employed interaction modeling to delineate sex-modified risks, leaving this area largely unexplored.

To address these gaps, we conducted a comprehensive analysis of sex-specific differences in mortality and cancer risk using a large, longitudinally followed Chinese pSD cohort. This study aimed to elucidate the impact of sex on clinical presentation, systemic involvement, and long-term outcomes, providing novel insights into sex-based prognostic disparities and informing more personalized approaches to disease monitoring and management.

## Patient and methods

### Study design and population

This ambispective cohort study was registered in the Clinical Trial Registry (ID: NCT06528197) and conducted in accordance with the Declaration of Helsinki. Ethical approval was granted by the Clinical Research Ethics Committee of the China-Japan Friendship Hospital (approval number: 2024-KY-173). Informed consent was waived for the retrospective component, and informed consent was obtained for the prospective follow-up phase. We used the STROBE reporting guidelines [26] to draft this manuscript and the STROBE reporting checklist [27] when editing, which are included in supplement 1.

We enrolled patients who visited China-Japan Friendship Hospital between January 2014 and September 2023. Eligible participants were those diagnosed with pSD according to either the 2002 American–European Consensus Group (AECG) criteria or the 2016 American College of Rheumatology/European League Against Rheumatism (ACR/EULAR) criteria, without fulfilling diagnostic criteria for other connective tissue diseases [28, 29]. The exclusion criteria were as follows: (1) missing key baseline data; (2) duplicate records; (3) refusal to participate in follow-up for primary outcome assessment; and (4) other conditions (e.g., cognitive impairment) that, in the investigators’ judgment, rendered a patient ineligible for enrollment.

### Data collection

Clinical data were collected at each patient’s first visit to the China-Japan Friendship Hospital. Outcomes were determined via telephone or face‒to-face follow-up and verified through electronic medical records. The demographic variables included sex, age at consultation, disease onset, and disease duration. The clinical manifestations recorded were xerostomia, xerophthalmia, fatigue, fever, rash, cough, dyspnea, lymphadenopathy, arthritis, rampant caries, and parotid enlargement. ILD was identified via high-resolution computed tomography (HRCT), independently reviewed by two experienced radiologists, and confirmed by clinicians. Splenomegaly was evaluated through abdominal ultrasonography or computed tomography. Disease activity was assessed via the EULAR Sjögren’s Syndrome Disease Activity Index (ESSDAI) [30].

All laboratory assessments were performed in the hospital’s central laboratory following standardized protocols. Hematologic abnormalities were defined as follows: leukopenia (< 3.5 × 10⁹/L), neutropenia (< 1.8 × 10⁹/L), lymphocytopenia (< 1.1 × 10⁹/L), thrombocytopenia (< 100 × 10⁹/L), and anemia (hemoglobin < 110 g/L in women or < 120 g/L in men). Biochemical abnormalities included elevated alanine aminotransferase (ALT > 40 U/L), aspartate aminotransferase (AST > 31 U/L), total bilirubin (TBIL > 23 μmol/L), γ-glutamyl transferase (GGT > 45 U/L in women or > 64 U/L in men), and creatinine (CREA > 97 μmol/L in women or > 106 μmol/L in men). Additional laboratory indicators included hypoalbuminemia (serum albumin < 35 g/L), hyponatremia (serum sodium < 137 mmol/L), hypokalemia (serum potassium < 3.5 mmol/L), and hypochloremia (serum chloride < 99 mmol/L). Hypergammaglobulinemia was defined as immunoglobulin (Ig) G > 16.2 g/L, IgA > 3.78 g/L, or IgM > 2.63 g/L. Hypocomplementemia was defined as complement component 3 (C3) < 0.70 g/L or component 4 (C4) < 0.16 g/L. Elevated inflammatory markers included C-reactive protein (CRP > 8 mg/L) and the erythrocyte sedimentation rate (ESR > 20 mm/h).

Autoantibodies were measured via commercial kits. Antinuclear antibody (ANA) titers were determined by indirect immunofluorescence in HEp-2 cells. Rheumatoid factor (RF) was detected via an immunoturbidimetric assay, with > 20 IU/mL considered positive. Anti-SSA and other autoantibodies were assessed by immunoblotting.

### Follow-up and outcomes

For this ambispective cohort, the index date was defined as the date of enrollment at our institution, at which time baseline clinical manifestations, comorbidities, laboratory parameters, and disease activity were systematically assessed. Disease duration was calculated as the time interval between the initial diagnosis of pSD and the index date. Follow-up time was calculated from the index date to the occurrence of outcomes or last follow-up. All eligible pSD patients were prospectively followed from the date of enrollment through telephone or in-person visits at 6-month intervals until death from any cause or study termination (February 2024). The primary endpoint was all-cause mortality. Cancer was defined as any newly diagnosed malignancy in patients without a prior cancer history at baseline. For deceased participants, survival time was defined as the interval between enrollment and death.

### Statistical analysis

Statistical analyses were conducted via SPSS v26.0 and R v4.2.3. The Shapiro–Wilk test was used to assess the normality of continuous variables. Categorical variables are summarized as frequencies and percentages and were compared via the *χ*^*2*^ test or Fisher’s exact test. Normally distributed continuous variables are expressed as the mean ± standard deviation (SD) and were compared with the independent-samples t test, whereas skewed data are presented as the median (interquartile range, *IQR*) and were compared with the Mann–Whitney U test.

Survival outcomes, including all-cause mortality and cancer incidence, were estimated via Kaplan–Meier curves and compared via the log-rank test. Standardized mortality ratios (SMRs) were computed as the ratio of observed to expected deaths, where expected deaths were derived from age- and sex-specific mortality rates in the 2019 national dataset of the Chinese Center for Disease Control and Prevention (http://www.stats.gov.cn/). The year 2019 was selected because it represents the midpoint of the study period (2014–2023) and provides a stable national mortality baseline. SMRs are reported with exact Poisson 95% confidence intervals (CIs). Cox proportional hazards regression was applied to identify factors independently associated with death and cancer. Variables with *P* < 0.05 in univariate analyses were entered into multivariable Cox models. Covariates that remained significant in the multivariable analysis were subsequently entered into a forward stepwise Cox model as a secondary, exploratory analysis to examine incremental changes in hazard ratios (HRs) for sex with sequential covariate adjustment. This approach was used to illustrate potential attenuation of the sex–outcome association rather than for variable selection or causal inference. Subgroup analyses were performed by sex to delineate sex-specific mortality risk factors, and interaction effects between sex and other covariates were further examined for multiple testing. Sex-stratified Cox proportional hazards models were performed to explore potential differences in prognostic patterns between men and women. Given the relatively small number of outcome events in certain sex-specific analyses, events-per-variable (EPV) ratios were explicitly evaluated prior to model construction. Parsimonious multivariable models were prespecified based on clinical relevance and results from univariable screening, rather than data-driven variable selection. When EPV was limited, particularly in male patients and in cancer-specific analyses, the number of covariates was restricted to maintain model stability. In these settings, penalized Cox regression using Firth’s correction was additionally performed as a sensitivity analysis to mitigate potential small-sample bias and separation. Estimates from Firth-corrected models were compared with those from standard Cox regression to assess the robustness of associations. Two-tailed tests with *P* set at 0.05 were considered significant. No missing data were detected.

Additive interaction was assessed using relative excess risk due to interaction (RERI), attributable proportion (AP), and synergy index (SI) to evaluate whether the joint effect of sex and specific clinical factors exceeded the sum of their individual effects. Unlike multiplicative interaction, additive interaction directly quantifies excess risk attributable to combined exposures and is therefore more informative for clinical risk stratification and identifying patient subgroups with disproportionately high absolute risk. A positive RERI or AP indicates a synergistic interaction on the additive scale, suggesting that patients exposed to both risk factors experience a greater-than-expected increase in risk.

## Results

**Fig. 1:**
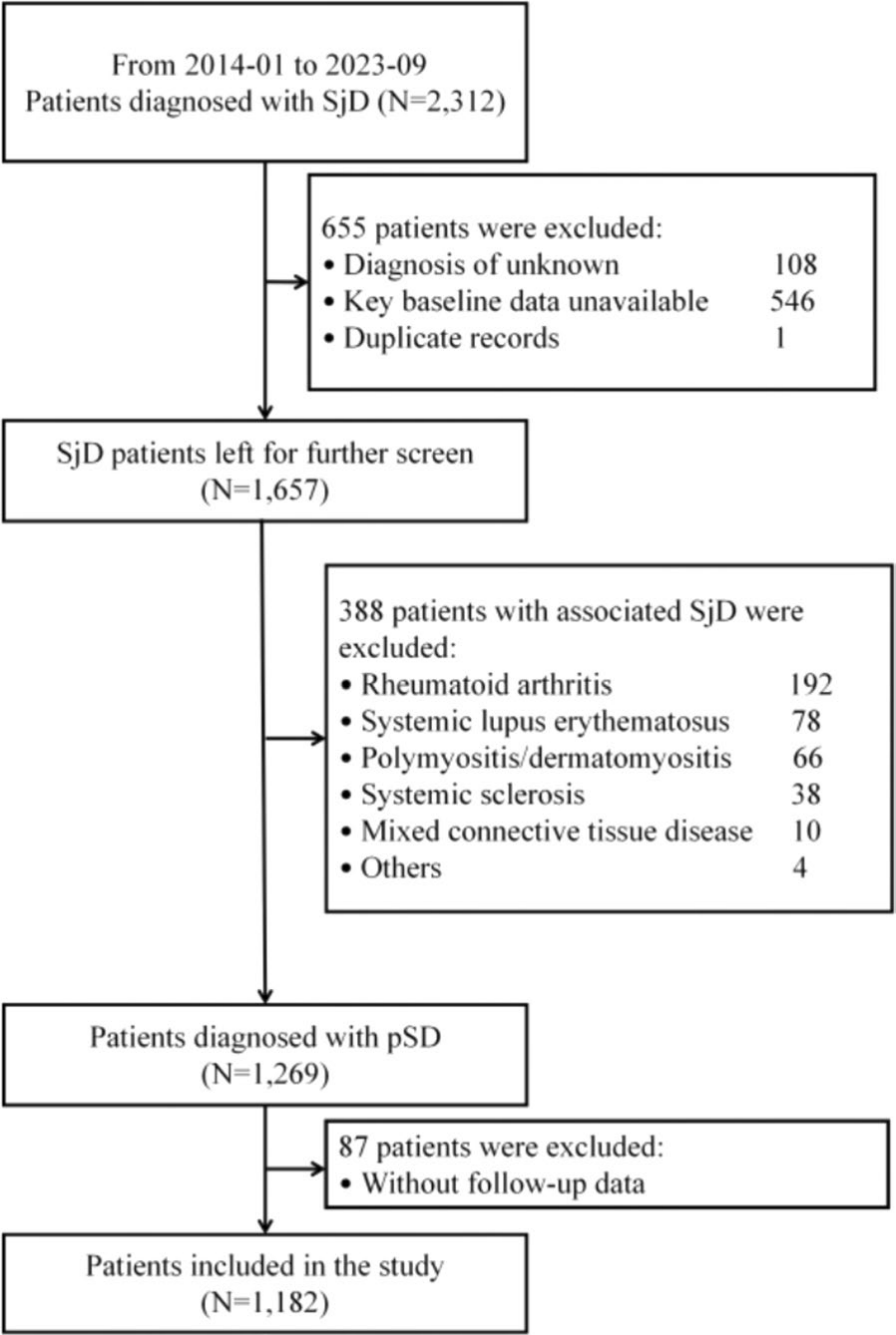
Flowchart for patient selection. SjD: Sjögren’s disease. pSD: primary Sjögren’s disease

A total of 2,312 patients diagnosed with Sjögren’s disease (SjD) at the China-Japan Friendship Hospital between January 2014 and September 2023 were initially screened. After excluding 1,043 patients, 1,269 patients with pSD were enrolled for follow-up, 87 of whom were lost to follow-up. Ultimately, 1,182 patients with pSD were included in the final analysis (Fig. 1).

### Baseline characteristics

Among the 1,182 patients with pSD, 1,025 were female (86.7%), and 157 were male (13.3%), revealing distinct sex-related clinical profiles. Compared with females, male patients presented at an older age, were diagnosed later, and had shorter disease durations. As summarized in Table 1, men presented lower frequencies of sicca symptoms (xerostomia and xerophthalmia), fatigue, and arthralgia but substantially higher rates of respiratory manifestations such as cough, dyspnea, and ILD. Similarly, ESSDAI scores were significantly higher in men.

**Table 1:** Sex-specific differences in clinical manifestations and comorbidities in patients with pSD

Variables	All(n = 1182)	Female(n = 1025)	Male(n = 157)	*P* value*
**Clinical characteristics**				
Age (years)	58 (49, 67)	58 (49, 66)	64 (54, 70)	< 0.001
Age at diagnosis (years)	52 (42.75, 62)	51 (42, 61)	61 (52.5, 68)	< 0.001
Disease duration (months)	36 (9, 96)	48 (12, 108)	12 (3, 48)	< 0.001
Xerostomia	992 (83.9)	883 (86.1)	109 (69.4)	< 0.001
Xerophthalmia	917 (77.6)	815 (79.5)	102 (65)	< 0.001
Fatigue	564 (47.7)	505 (49.3)	59 (37.6)	0.006
Fever	137 (11.6)	112 (10.9)	25 (15.9)	0.069
Purpuric rash	87 (7.4)	80 (7.8)	7 (4.5)	0.135
Arthralgia	394 (33.3)	365 (35.6)	29 (18.5)	< 0.001
Arthritis	101 (8.5)	93 (9.1)	8 (5.1)	0.097
Dyspnea	336 (28.4)	262 (25.6)	74 (47.1)	< 0.001
Cough	394 (33.3)	308 (30)	86 (54.8)	< 0.001
Rampant caries	340 (28.8)	320 (31.2)	20 (12.7)	< 0.001
Lymphadenopathy	212 (17.9)	185 (18)	27 (17.2)	0.796
Parotid enlargement	87 (7.4)	76 (7.4)	11 (7)	0.855
Splenomegaly	54 (4.6)	48 (4.7)	6 (3.8)	0.63
Hemorrhage	74 (6.3)	66 (6.4)	8 (5.1)	0.518
ILD	372 (31.5)	282 (27.5)	90 (57.3)	< 0.001
ESSDAI score	7 (3, 14)	6 (3, 13)	10 (3, 16.5)	0.017
**Comorbidities**				
Hypertension	302 (25.5)	252 (24.6)	50 (31.8)	0.052
Gastroesophageal reflux disease	284 (24.0)	235 (22.9)	49 (31.2)	0.024
Osteoporosis	273 (23.1)	242 (23.6)	31 (19.7)	0.285
Dyslipidemia	217 (18.4)	189 (18.4)	28 (17.8)	0.855
Atherosclerosis	167 (14.1)	135 (13.2)	32 (20.4)	0.016
Hepatic steatosis	155 (13.1)	132 (12.9)	23 (14.6)	0.54
Osteoarthritis	137 (11.6)	131 (12.8)	6 (3.8)	0.001
Hypothyroidism	126 (10.7)	120 (11.7)	6 (3.8)	0.003
Type 2 diabetes mellitus	108 (9.1)	80 (7.8)	28 (17.8)	< 0.001
Pulmonary infection	104 (8.8)	84 (8.2)	20 (12.7)	0.061
Ischemic stroke	104 (8.8)	88 (8.6)	16 (10.2)	0.508
Coronary artery disease	97 (8.2)	75 (7.3)	22 (14)	0.004
Liver cyst	84 (7.1)	74 (7.2)	10 (6.4)	0.699
Cholelithiasis	67 (5.7)	54 (5.3)	13 (8.3)	0.129
Primary biliary cholangitis	61 (5.2)	61 (6.0)	0 (0.0)	0.002
Hyperuricemia	45 (3.8)	32 (3.1)	13 (8.3)	0.002
Respiratory failure	42 (3.6)	29 (2.8)	13 (8.3)	0.001
Heart failure	41 (3.5)	35 (3.4)	6 (3.8)	0.795
Chronic obstructive pulmonary disease	36 (3.0)	26 (2.5)	10 (6.4)	0.009
Cancer	23 (1.9)	18 (1.8)	5 (3.2)	0.228

Data are presented as the median (IQR) or n (%)

Comparative analyses were performed between male and female patients with primary Sjögren’s disease

*P values were calculated via the *χ*^*2*^ test or Fisher’s exact test for categorical variables and the Mann‒Whitney U test for continuous variables

ILD: interstitial lung disease. ESSDAI: European Alliance of Associations for Rheumatology (EULAR) Sjögren’s Syndrome Disease Activity Index score

The comorbidity spectrum also differed by sex: men were more likely to have type 2 diabetes mellitus, respiratory failure, chronic obstructive pulmonary disease, coronary artery disease, atherosclerosis, gastroesophageal reflux disease, and hyperuricemia, whereas hypothyroidism, primary biliary cholangitis, and osteoarthritis predominated among women. No significant sex-related differences were observed in the incidence of hypertension, osteoporosis, or malignancy. Collectively, these findings indicate that male pSD patients tend to have later disease onset, shorter disease duration, milder sicca features, and greater respiratory involvement and comorbidity burden.

Distinct sex-specific laboratory patterns were evident (Table 2). Compared with men, women presented greater frequencies of leukopenia (20.2% vs 8.3%, *P* < 0.001), neutropenia (17.9% vs 7.0%, *P* = 0.001), elevated AST (16.8% vs 7.6%, *P* = 0.003), hypokalemia (9.5% vs 3.8%, *P* = 0.02), hypergammaglobulinemia (58.9% vs 45.9%, *P* = 0.002), hyper-IgG (48.1% vs 33.8%, *P* = 0.001), elevated ESR (52.1% vs 43.3%, *P* = 0.040), and hypocomplementemia (44.2% vs 28.0%, *P* < 0.001). In contrast, men presented significantly higher rates of anemia (70.1% vs 30.8%, *P* < 0.001), hypoalbuminemia (10.2% vs 19.7%, *P* = 0.001) and elevated CRP levels (38.9% vs 22.9%, *P* < 0.001), suggesting a stronger systemic inflammatory response. Autoantibody positivity, including ANA (65.1% vs 42.0%, *P* < 0.001), RF (47.3% vs 36.3%, *P* = 0.01), anti-SSA (75.5% vs 43.9%, *P* < 0.001), anti-Ro52 (60.7% vs 36.3%, *P* < 0.001), anti-SSB (29.2% vs 21.0%, *P* = 0.034), and anti-CENP-B (9.6% vs 3.2%, *P* = 0.008) positivity, was consistently greater in women than in men.

**Table 2:** Sex-specific differences in the laboratory features of patients with pSD

Laboratory features	All(n = 1182)	Female(n = 1025)	Male(n = 157)	*P* value*
Leukopenia	220 (18.6)	207 (20.2)	13 (8.3)	< 0.001
Neutropenia	194 (16.4)	183 (17.9)	11 (7.0)	0.001
Lymphocytopenia	300 (25.4)	265 (25.9)	35 (22.3)	0.340
Thrombocytopenia	174 (14.7)	159 (15.5)	15 (9.6)	0.050
Anemia	426 (36.0)	316 (30.8)	110 (70.1)	< 0.001
Elevated ALT	135 (11.4)	117 (11.4)	18 (11.5)	0.985
Elevated AST	184 (15.6)	172 (16.8)	12 (7.6)	0.003
Elevated TBIL	45 (3.8)	39 (3.8)	6 (3.8)	0.992
Elevated GGT	208 (17.6)	185 (18)	23 (14.6)	0.298
Elevated creatinine	70 (5.9)	58 (5.7)	12 (7.6)	0.327
Hypoalbuminemia	136 (11.5)	105 (10.2)	31 (19.7)	0.001
Hyponatremia	137 (11.6)	116 (11.3)	21 (13.4)	0.453
Hypokalemia	103 (8.7)	97 (9.5)	6 (3.8)	0.020
Hypochloremia	30 (2.5)	25 (2.4)	5 (3.2)	0.580
Hypergammaglobulinemia	676 (57.2)	604 (58.9)	72 (45.9)	0.002
Hyper-IgG (> 16.2 g/L)	546 (46.2)	493 (48.1)	53 (33.8)	0.001
Hyper-IgA (> 3.78 g/L)	306 (25.9)	270 (26.3)	36 (22.9)	0.363
Hyper-IgM (> 2.63 g/L)	88 (7.4)	81 (7.9)	7 (4.5)	0.126
Hypocomplementemia	497 (42.0)	453 (44.2)	44 (28.0)	< 0.001
Low complement C3 (< 0.70 g/L)	261 (22.1)	237 (23.1)	24 (15.3)	0.028
Low complement C4(< 0.16 g/L)	409 (34.6)	374 (36.5)	35 (22.3)	< 0.001
Elevated CRP (> 8 mg/L)	296 (25.0)	235 (22.9)	61 (38.9)	< 0.001
Elevated ESR (> 20 mm/h)	602 (50.9)	534 (52.1)	68 (43.3)	0.040
ANA titers ≥ 1:160	733 (62.0)	667 (65.1)	66 (42.0)	< 0.001
Positive RF	542 (45.9)	485 (47.3)	57 (36.3)	0.010
Positive anti-SSA	843 (71.3)	774 (75.5)	69 (43.9)	< 0.001
Positive anti-Ro-52	679 (57.4)	622 (60.7)	57 (36.3)	< 0.001
Positive anti-SSB	332 (28.1)	299 (29.2)	33 (21.0)	0.034
Positive anti-RNP	84 (7.1)	78 (7.6)	6 (3.8)	0.085
Positive anti-CENP-B	103 (8.7)	98 (9.6)	5 (3.2)	0.008
Positive anti-AMA-M2	103 (8.7)	96 (9.4)	7 (4.5)	0.042

Data are presented as the median (IQR) or n (%)

Comparative analyses were conducted between male and female patients with primary Sjögren’s disease

*P values were calculated via the *χ*^*2*^ test or Fisher’s exact test for categorical variables and the Mann‒Whitney U test for continuous variables

ALT: alanine aminotransferase. AST: aspartate aminotransferase. TBIL: total bilirubin. GGT: γ-glutamyl transferase. Hyper-IgG: Hypergammaglobulinemia with elevated immunoglobulin G. Hyper-IgA: Hypergammaglobulinemia with elevated immunoglobulin A. Hyper-IgM: Hypergammaglobulinemia with elevated immunoglobulin M. Elevated CRP: Elevated C-reactive protein. Elevated ESR: elevated erythrocyte sedimentation rate

### Survival and cancer outcomes

The mean follow-up duration was four years (range: 0.08–12 years). During this period, 125 patients (10.6%) died, corresponding to an SMR of 2.87 (95% CI: 2.39–3.42), indicating significantly elevated mortality compared with that of the general population. The excess mortality was especially pronounced in men, with a 25.5% death rate and an SMR of 4.79 (95% CI: 3.42–6.52), which was markedly higher than that in women (8.3%, SMR = 2.42, 95% CI: 1.93–2.99). Infection was the leading cause of death (44.0%), followed by cancer (13.6%), respiratory failure (11.2%), and cardiovascular events (8.8%). Compared with women, men had higher proportions of infection- and respiratory-related deaths (10.8% vs 3.7%, *P* < 0.001; 3.2% vs 0.9%, *P* = 0.028, respectively), as did women, as well as a higher proportion of malignancy-related deaths (4.5% vs 1.0%, *P* = 0.004; Supplementary Table S1).

During follow-up, 33 patients (2.8%) developed malignancies, with a significantly greater incidence in men than in women (6.4% vs 2.2%, *P* = 0.008). The most common cancer types were respiratory (30.3%) and digestive (21.2%) system malignancies, followed by thyroid and gynecologic/breast cancers. Hematologic malignancies were rare (0.3%) and occurred exclusively in female patients. Given the limited number of cancer events, the distribution of cancer subtypes is provided in the Supplementary Table 1 for descriptive purposes only.

**Fig. 2:**
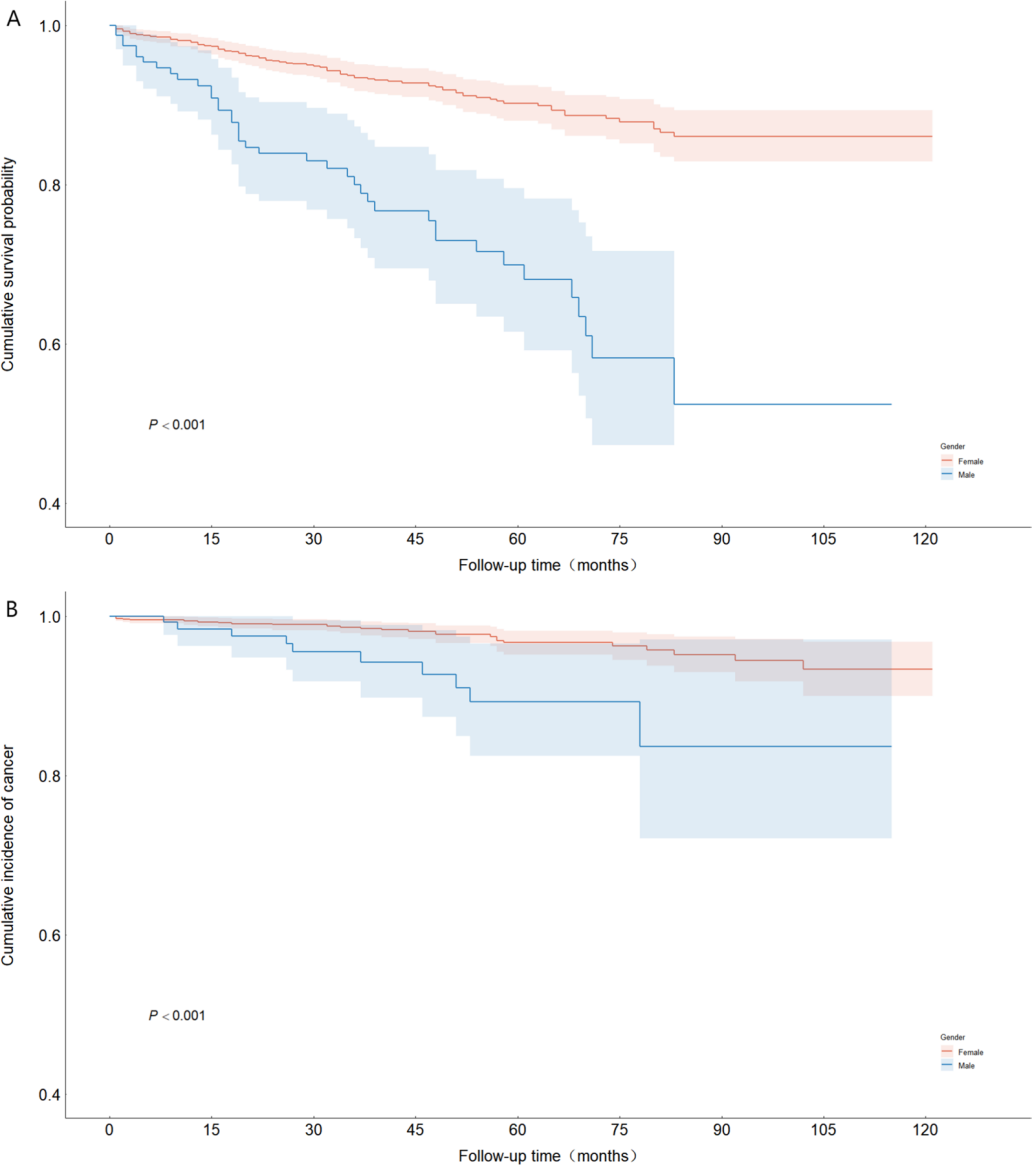
Kaplan‒Meier curves according to sex. (**A**) Kaplan‒Meier curves for all-cause death in patients with pSD stratified by sex. (**B**) Kaplan‒Meier curves for cancer patients with pSD stratified by sex

Kaplan–Meier analyses confirmed pronounced sex-specific disparities in prognosis: male patients demonstrated a significantly greater cumulative risk of both all-cause mortality (Fig. 2A) and cancer (Fig. 2B) than female patients did (log-rank tests, *P* < 0.001).

### Factors associated with death and cancer in the overall cohort

#### Death

**Fig. 3:**
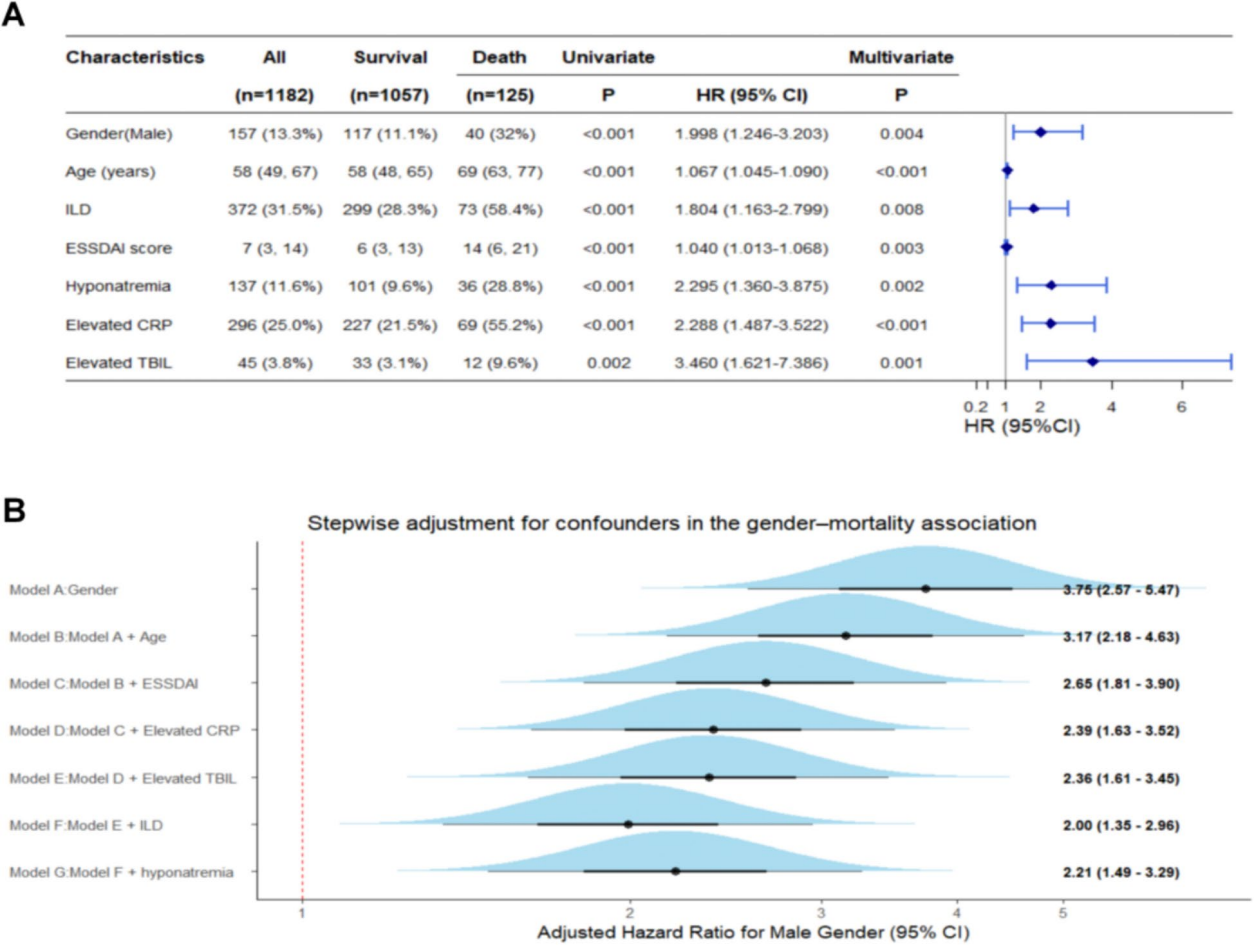
Sex-specific prognostic factors and stepwise Cox regression for death in patients with pSD. (**A**) Forest plot showing univariate and multivariate Cox regression analyses of prognostic factors for all-cause death in the total cohort (n = 1,182). Male sex, older age, ILD, higher ESSDAI score, hyponatremia, elevated CRP, and elevated TBIL were identified as independent predictors of mortality. HRs with 95% CIs are displayed, and significant results are indicated in blue. (**B**) Forward stepwise Cox proportional hazards model illustrating the sequential adjustment for confounders in the sex‒death association. The adjusted HR for male sex remained significant after stepwise inclusion of covariates (from 3.75 to 2.21). ILD: interstitial lung disease. ESSDAI: European Alliance of Associations for Rheumatology (EULAR) Sjögren’s Syndrome Disease Activity Index score. CRP: C-reactive protein. TBIL: total bilirubin. HR: hazard ratio. 95% CI: 95% confidence interval

Among the 1,182 patients with pSD, 125 patients (10.6%) died, while 1,057 patients survived during follow-up. Univariable Cox regression identified several clinical variables significantly associated with all-cause mortality. In the multivariable model incorporating all variables with *P* < 0.05, factors independently associated with death included male sex (HR = 1.998, 95% CI: 1.246–3.203, *P* = 0.004), advanced age (HR = 1.067, 95% CI: 1.045–1.090, *P* < 0.001), ILD (HR = 1.804, 95% CI: 1.163–2.799, *P* = 0.008), elevated ESSDAI score (HR = 1.040, 95% CI: 1.013–1.068, *P* = 0.003), hyponatremia (HR = 2.295, 95% CI: 1.360–3.875, *P* = 0.002), elevated CRP (HR = 2.288, 95% CI: 1.487–3.522, *P* < 0.001), and elevated TBIL (HR = 3.460, 95% CI: 1.621–7.386, *P* = 0.001; Fig. 3A; full data in Supplementary Table S2).

Stepwise Cox regression illustrated the progressive attenuation of the HR for male sex with respect to mortality, as significant confounders were sequentially adjusted. Although the HR decreased from 3.75 to 2.21, it remained significantly elevated (Fig. 3B). Age- and sex-stratified analyses revealed the progressive accumulation of high-risk features with advancing age in both sexes, although this trend was markedly stronger in men. Among males aged ≥ 50 years, middle–high ESSDAI scores, ILD, elevated CRP, hyponatremia, and elevated TBIL were more prevalent and closely associated with increased mortality. In contrast, women exhibited a more balanced distribution of prognostic factors; despite age-related increases in the ESSDAI and ILD, their overall mortality risk remained lower (Supplementary Fig. S1A).

To assess the robustness of the association between sex and all-cause mortality, we further performed a prespecified fully adjusted Cox regression model in which all clinically relevant covariates were entered simultaneously without stepwise selection. In this fully adjusted model, male sex remained independently associated with a higher risk of all-cause death, with the direction and magnitude of the HR largely consistent with the primary analyses (Supplementary Table S3).

#### Cancer

**Fig. 4:**
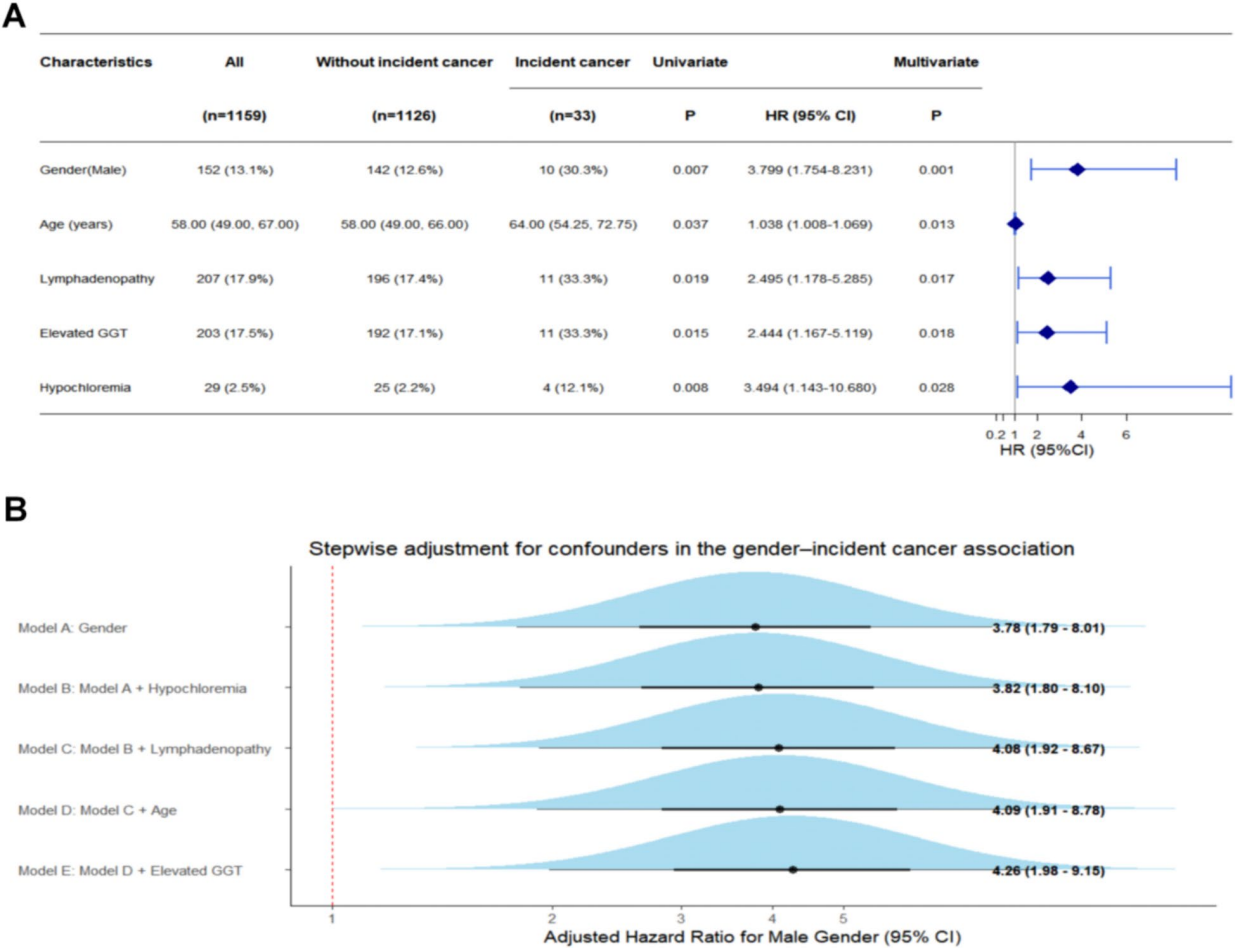
Sex-specific prognostic factors and stepwise Cox regression for cancer in patients with pSD. (**A**) Forest plot showing univariate and multivariate Cox regression analyses of prognostic factors for cancer in the total cohort (n = 1,159). Male sex, lymphadenopathy, lymphocytopenia, elevated GGT, and liver cysts were identified as independent prognostic factors for cancer development. HRs with 95% CIs are shown; significant variables are highlighted in blue. (**B**) Forward stepwise Cox proportional hazards model illustrating sequential adjustment for confounders in the sex‒cancer association. The adjusted HR for male sex remained significant after stepwise inclusion of covariates (from 3.69 to 4.01). GGT: γ-glutamyl transpeptidase. HR: hazard ratio. 95% CI: 95% confidence interval

For cancer outcomes, 33 (2.8%) of the 1,159 pSD patients developed malignancies during follow-up, whereas 1,126 did not. Multivariate Cox regression identified male sex (HR = 3.799, 95% CI: 1.754–8.231, *P* = 0.001), advanced age (HR = 1.038, 95% CI: 1.008–1.069, *P* = 0.013), lymphadenopathy (HR = 2.42, 95% CI: 1.16–5.09, *P* = 0.019), elevated GGT (HR = 2.444, 95% CI: 1.167–5.119, *P* = 0.018), and hypochloremia (HR = 3.494, 95% CI: 1.143–10.68, *P* = 0.028) were independently associated with cancer (Fig. 4A; full data in Supplementary Table S4).

Stepwise Cox regression for cancer risk illustrated the impact of sequential covariate adjustment on the HR for male sex. Although the HR slightly fluctuated during the process, it increased from an initial value of 3.78 to a final value of 4.26 in the fully adjusted model (Fig. 4B). Stratified visualization revealed distinct sex- and age-specific patterns: both sexes presented an accumulation of factors associated with cancer, notably elevated GGT and lymphadenopathy, predominantly between 40–79 years of age. However, female patients presented clustered prognostic factors, whereas men aged 50–79 years presented a broader accumulation of multiple high-risk features (Supplementary Fig. S1B).

Similarly, a prespecified fully adjusted Cox regression model was constructed for cancer outcomes, including all clinically relevant covariates simultaneously. The association between male sex and cancer remained consistent in direction and magnitude after full adjustment, supporting the robustness of the sex–cancer association (Supplementary Table S5).

### Sex-stratified prognostic analyses

To further delineate sex-specific prognostic patterns, subgroup analyses were performed separately for men and women to identify factors independently associated with mortality and cancer. Among the 157 male patients with pSD, 40 (25.5%) died during follow-up, whereas among the 1,025 female patients, 85 (8.3%) died.

In male patients, initial multivariate Cox regression revealed that ILD (HR = 7.35, 95% CI: 2.15–25.12, *P* = 0.001), pulmonary infection (HR = 4.90, 95% CI: 1.83–13.06, *P* = 0.002), and ischemic stroke (HR = 2.52, 95% CI: 1.06–5.99, *P* = 0.037) were independently associated with death, whereas age and disease duration lost significance after adjustment (Table 3; full data in Supplementary Table S6). Given the limited number of death events in men, EPV considerations were explicity addressed. A clinically prespecified parsimonious multivariable Cox model was constructed (EPV = 10.0), including age, ILD, pulmonary infection, and ischemic stroke. In this model, age (HR = 1.07, 95% CI: 1.02–1.11, P = 0.005), ILD (HR = 7.22, 95% CI: 2.75–18.92, P < 0.001), pulmonary infection (HR = 4.65, 95% CI: 2.06–10.53, P < 0.001), and ischemic stroke (HR = 2.75, 95% CI: 1.23–6.12, P = 0.013) remained independently associated with all-cause death. A sensitivity analysis using penalized Cox regression with Firth’s correction yielded directionally consistent estimates for all covariates (Supplementary Table S7).

**Table 3:** Sex-stratified univariate and multivariate Cox regression analyses of factors associated with death in patients with pSD

Subgroups	Variables	All	Survival	Death	Univariate analysis	Multivariate analysis	
					*P* value*	HR (95% CI)	*P* value†
**Male**							
	Age (years)	64.0 (54.0, 70.0)	61.0 (50.0, 68.0)	68.0 (64.0, 74.0)	< 0.001	1.043 (0.995, 1.092)	0.078
	Disease duration (months)	12.0 (3.0,48.0)	12.0 (3.0,48.0)	4.5 (1.0,36.0)	0.034	1.001 (0.991, 1.011)	0.835
	ILD	90 (57.3)	56 (47.9)	34 (85.0)	< 0.001	7.350 (2.150, 25.121)	0.001
	Pulmonary infection	20 (12.7)	8 (6.8)	12 (30.0)	< 0.001	4.895 (1.834, 13.062)	0.002
	Ischemic stroke	16 (10.2)	8 (6.8)	8 (20.0)	0.018	2.515 (1.057, 5.987)	0.037
**Female**							
	Age (years)	58 (49.0, 66.0)	57 (48, 65)	69 (61.5, 77.5)	< 0.001	1.077 (1.051, 1.105)	< 0.001
	Disease duration (months)	48 (12, 108)	48 (12, 108)	36 (5.5, 120)	0.589	/	/
	Low C3	237(23.1)	210(22.3)	27(31.8)	0.048	2.057 (1.157, 3.657)	0.014
	Elevated CRP (> 8 mg/L)	235(22.9)	189(20.1)	46(54.1)	< 0.001	2.491 (1.391, 4.461)	0.002
	Elevated TBIL	39(3.8)	28(3)	11(12.9)	< 0.001	4.255 (1.675, 10.807)	0.002

Data are presented as the median (IQR) or n (%). Comparative analyses were conducted between the survival and death groups of patients with primary Sjögren’s disease

* Calculated via the *χ*^*2*^ test or Fisher’s exact test for categorical variables and the Mann‒Whitney U test for continuous variables

† Variables with *P* < 0.05 in the univariate analysis were included in the multivariable Cox regression model

ILD: interstitial lung disease. C3: component 3; CRP: C-reactive protein. TBIL: total bilirubin. HR: hazard ratio. CI: confidence interval

In contrast, the female subgroup demonstrated a distinct risk profile. Multivariable Cox regression showed that older age (HR = 1.08, 95% CI: 1.05–1.11, *P* < 0.001), low complement C3 (HR = 2.06, 95% CI: 1.16–3.66, *P* = 0.014), elevated CRP (HR = 2.49, 95% CI: 1.39–4.46, *P* = 0.002), and elevated TBIL (HR = 4.26, 95% CI: 1.68–10.81, *P* = 0.002) were independently associated with increased mortality (Table 3; full data in Supplementary Table S8). Penalized Cox regression using Firth’s correction confirmed the robustness of these associations (EPV = 21.25; Supplementary Table S7).

With respect to cancer, 10 of 152 male patients (6.6%) developed malignancies, whereas 26 of 1,007 female patients (2.6%) developed malignancies. Among male patients, 10 malignancy events occurred during follow-up. Due to the limited number of cancer events, no stable independent prognostic factors were identified in male-specific multivariable analyses. In age-adjusted analyses, neither age nor disease activity was significantly associated with cancer risk (Table 4; full data in Supplementary Table S9). A sensitivity analysis using penalized Cox regression with Firth’s correction yielded similar null results, indicating no robust independent predictors of malignancy in men (EPV = 5.0; Supplementary Table S10). In women, however, multivariable Cox regression identified longer disease duration (HR = 1.01, 95% CI: 1.00–1.01, *P* = 0.002), lymphadenopathy (HR = 4.75, 95% CI: 1.99–11.33, *P* = 0.001), lymphocytopenia (HR = 2.60, 95% CI: 1.09–6.18, *P* = 0.031), elevated TBIL (HR = 4.27, 95% CI: 1.09–16.73, *P* = 0.037), and hypochloremia (HR = 3.68, 95% CI: 1.03–13.23, *P* = 0.046) independently associated with cancer (Table 4; full data in Supplementary Table S11). Given the limited number of cancer events, penalized Cox regression using Firth’s correction was performed as a sensitivity analysis and yielded directionally consistent estimates (EPV = 6.5; Supplementary Table S10).

**Table 4:** Univariate and multivariate Cox regression analyses of factor associated with cancer in the female subgroup of patients with pSD

Subgroups	Variables	All	Without cancer	With cancer	Univariate analysis	Multivariate analysis	
					*P* value*	HR (95% CI)	*P* value†
**Male**							
	Age (years)	64.0 (54.0, 70.0)	63.5 (53.75, 69.0)	70.0 (53.8, 73.3)	0.181	/	/
	Disease duration (months)	12.0 (3.0, 48.0)	12.0 (3.0, 48.0)	24.0 (1.0, 27.0)	0.751	/	/
**Female**							
	Age (years)	58 (49,66)	58 (48,66)	61 (54,70)	0.127	/	/
	Disease duration (months)	48 (12,108)	48 (12,108)	96 (36,156)	0.016	1.005 (1.002,1.008)	0.002
	Lymphadenopathy	182 (18.1)	172 (17.5)	10 (43.5)	0.004	4.748 (1.989,11.334)	< 0.001
	Lymphocytopenia	259 (25.7)	248 (25.2)	11 (47.8)	0.014	2.597 (1.091,6.180)	0.031
	Elevated TBIL	37 (3.7)	34 (3.5)	3 (13.0)	0.049	4.27 (1.090,16.726)	0.037
	Hypochloremia	25 (2.5)	22 (2.2)	3 (13.0)	0.017	3.684 (1.026,13.231)	0.046

Data are presented as the median (IQR) or n (%). Comparative analyses were conducted between the groups of patients with primary Sjögren’s disease with and without cancer

* Calculated via the *χ*^*2*^ test or Fisher’s exact test for categorical variables and the Mann‒Whitney U test for continuous variables

† Variables with *P* < 0.05 in the univariate analysis were included in the multivariable Cox regression model

TBIL: total bilirubin. HR: hazard ratio. CI: confidence interval

### Interaction analysis between sex and key factors associated with outcomes

Interaction analyses for all-cause death revealed significant additive interactions between sex and several prognostic factors. Notably, the ESSDAI score showed a strong positive interaction with sex (RERI = 5.57, AP = 0.52, SI = 2.37), indicating that men with greater disease activity faced a disproportionately greater mortality risk. Similarly, ILD demonstrated a significant additive interaction with sex (RERI = 6.13, AP = 0.66, SI = 3.81), suggesting that the association between ILD and mortality was modified by sex, with a stronger effect observed in men.

In contrast, elevated CRP, hyponatremia, elevated TBIL, and age exhibited no meaningful additive interactions with sex. These results highlight sex as a critical effect modifier, with male patients experiencing high disease activity or concomitant ILD showing a markedly increased mortality risk (Table 5). Stratification of patients by ESSDAI-defined disease activity (low, moderate, and high) revealed a significant, stepwise increase in mortality risk: compared with the low ESSDAI group, the HRs were 1.82 (95% CI: 1.08–3.08, *P* = 0.025) for the moderate-activity group and 4.93 (95% CI: 3.04–7.99, *P* < 0.001) for the high-activity group. Although age did not demonstrate a significant interaction with sex, its paramount clinical importance prompted a stratified analysis. Compared with those of the < 50 years age group, the HRs for mortality were 4.41 (95% CI: 1.86–10.49) for the 50–64 years age group, 14.66 (95% CI: 6.26–34.29) for the 65–74 years age group, and 32.98 (95% CI: 13.87–78.41) for the ≥ 75 years age group (all *P* < 0.005) (Table 6).

**Table 5:** Interaction analysis between gender and clinical factors associated with death in patients with pSD

Variables	Multiplicative interaction†	Additive interaction‡		
	OR (95% CI)	RERI	AP	SI
Gender*ESSDAI score	0.992 (0.957, 1.029)	5.57 (0.58, 10.56)	0.52 (0.22, 0.83)	2.37 (1.09, 5.18)
Gender*Elevated CRP	0.511 (0.239, 1.091)	1.87 (−2.71, 6.46)	0.20 (−0.22, 0.61)	1.28 (0.71, 2.31)
Gender*Elevated TBIL	0.128 (0.016, 1.027)	−5.70 (−12.50, 1.10)	−1.97(−7.91, 3.97)	0.25 (0.01, 5.16)
Gender*ILD	2.28 (0.866, 6.004)	6.13 (2.32, 9.94)	0.66 (0.46, 0.86)	3.81 (1.69, 8.60)
Gender*Hyponatremia	0.456 (0.186, 1.119)	1.02 (−5.47, 7.51)	0.12 (−0.55, 0.78)	1.15 (0.49, 2.69)
Gender*Age	1 (0.957, 1.044)	0.22 (−0.62, 1.07)	0.06 (0.01, 1.14)	1.10 (1.05, 1.14)

The data are presented as ORs with 95% CIs for multiplicative interactions and as RERIs, APs, and SIs for additive interactions

† Multiplicative interactions are expressed as ORs with 95% CIs. For multiplicative interactions, OR > 1 indicates a positive interaction, and OR < 1 indicates a negative interaction, with the 95% CI crossing 1 interpreted as nonsignificant

‡ Additive interactions were assessed by calculating the RERI, AP, and SI. For additive interactions, RERI > 0, AP > 0, or SI > 1 indicate positive (synergistic) interactions, whereas intervals crossing 0 (for RERI and AP) or 1 (for SI) suggest no additive interaction

RERI: relative excess risk due to interaction. AP: attributable proportion. SI: synergy index. ESSDAI: European Alliance of Associations for Rheumatology (EULAR) Sjögren’s Syndrome Disease Activity Index score. CRP: C-reactive protein. TBIL: total bilirubin. ILD: interstitial lung disease. OR: odds ratio. CI: confidence interval

**Table 6:** Stratified Cox Regression Analysis of Mortality by Age Group and ESSDAI Level in patients with pSD

Variables	Subgroups	HR(95% CI)	*P* value*
**Age (years)**			
	< 50	1.00 (Reference)	< 0.001
	50–64	4.41 (1.86–10.49)	0.001
	65–74	14.66 (6.26–34.29)	< 0.001
	≥ 75	32.98 (13.87–78.41)	< 0.001
**ESSDAI score**			
	< 5	1.00 (Reference)	< 0.001
	5–13	1.82 (1.08–3.08)	0.025
	> 13	4.93 (3.04–7.99)	< 0.001

Data are presented as the median (IQR). Comparative analyses were conducted between the reference group and subgroups of patients with primary Sjögren’s disease

* Calculated via the multivariable Cox regression model

ESSDAI: European Alliance of Associations for Rheumatology (EULAR) Sjögren’s Syndrome Disease Activity Index score. HR: hazard ratio. CI: confidence interval

For cancer outcomes, neither multiplicative nor additive interactions were detected between sex and other independent prognostic factors, including lymphadenopathy, older age, hypochloremia, and elevated GGT (Table 7). This finding indicates that sex did not affect the effects of these variables on cancer risk.

**Table 7:** Interaction analysis between gender and clinical factors associated with cancer in patients with pSD

Variables	Multiplicative interaction†		Additive interaction‡		
	OR (95% CI)		RERI	AP	SI
Gender*Lymphadenopathy	0.165 (0.018,1.525)		−5.06 (−15.26,5.15)	−1.18 (−5.60,3.24)	0.39 (0.03,5.42)
Gender*Elevated GGT	1.76 (0.375,8.272)		7.51 (−4.95,19.98)	0.64 (0.20,1.08)	3.28 (0.79,13.68)
Gender*Age	0.991 (0.93,1.056)		0.15 (−0.37,0.68)	0.02 (−0.01,0.06)	1.03 (0.97,1.09)
Gender*Hypochloremia	0.811 (0.074,8.943)		12.26 (−32.43,56.95)	0.55 (−0.40,1.51)	2.38 (0.25,22.86)

The data are presented as ORs with 95% CIs for multiplicative interactions and RERIs, APs, and SIs for additive interactions

† Multiplicative interactions are expressed as ORs with 95% CIs. For multiplicative interactions, OR > 1 indicates a positive interaction, and OR < 1 indicates a negative interaction, with the 95% CI crossing 1 interpreted as nonsignificant

‡ Additive interactions were assessed by calculating the RERI, AP, and SI. For additive interactions, RERI > 0, AP > 0, or SI > 1 indicate positive (synergistic) interactions, whereas intervals crossing 0 (for RERI and AP) or 1 (for SI) suggest no additive interaction

RERI: relative excess risk due to interaction. AP: attributable proportion. SI: synergy index. GGT: γ-glutamyl transferase. OR: odds ratio. CI: confidence interval

**Fig. 5:**
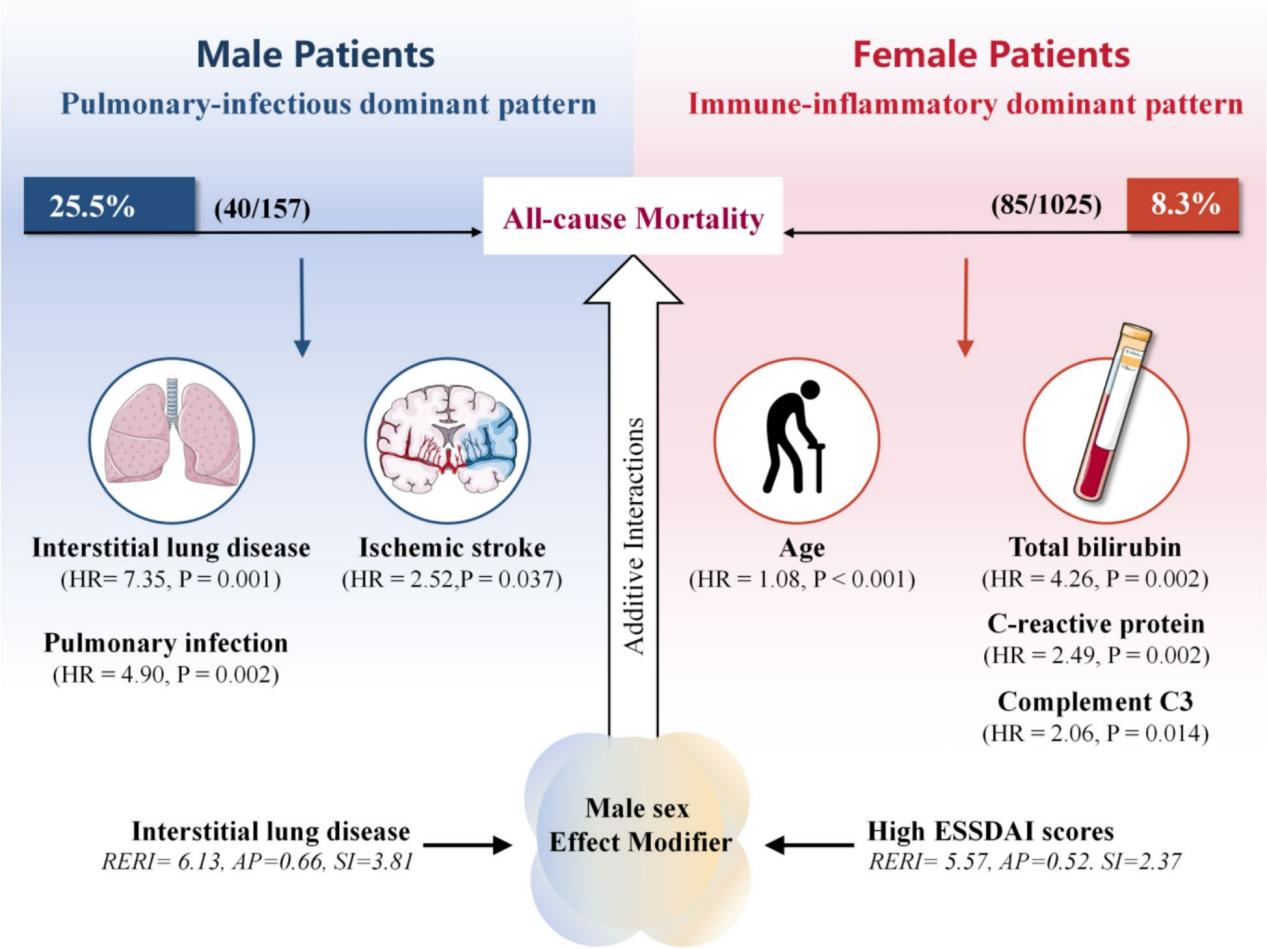
Sex Differences in Prognostic Pathways and Additive Interactions in pSD

To facilitate clinical interpretation of these findings, we summarized the sex-specific prognostic pathways in a schematic diagram (Fig. 5).

The upper panels summarize the distinct mortality-related risk profiles observed in male and female patients. Male patients (left, blue panel) exhibit a “Pulmonary-infectious dominant pattern” with a markedly higher mortality rate (25.5%), driven predominantly by interstitial lung disease (ILD), pulmonary infection, and ischemic stroke. In contrast, female patients (right, red panel) demonstrate an “Immune-inflammatory dominant pattern” (8.3% mortality), where risk is primarily associated with older age, systemic inflammation (elevated C-reactive protein), immune dysregulation (low complement C3), and metabolic abnormalities. Hazard Ratios (HR) are derived from sex-stratified multivariable Cox regression.

The lower panel details the additive interaction analysis. The central element represents male sex as a critical effect modifier. Red text with solid arrows identifies factors (ILD and High ESSDAI scores) that exhibit a synergistic interaction with male sex, significantly amplifying mortality risk beyond the sum of individual effects. This is quantified by the Relative Excess Risk due to Interaction (RERI), Attributable Proportion (AP), and Synergy Index (SI).

## Discussion

This cohort of 1,182 patients with pSD revealed pronounced sex-related differences across the clinical, serological, and prognostic domains. Male patients were older at diagnosis, had a shorter disease duration, and presented fewer sicca symptoms but greater systemic activity and comorbidity burden—particularly respiratory involvement, such as ILD, respiratory failure, and chronic obstructive pulmonary disease. In contrast, female patients demonstrated stronger autoimmune serological responses, including higher frequencies of ANA, anti-SSA/Ro52 positivity, hypergammaglobulinemia, and hypocomplementemia. Collectively, these findings demonstrate distinct clinical phenotypes between men and women with pSD.

Sex has also emerged as a critical determinant of long-term outcomes. Over a mean follow-up of 4 years in our study, the overall mortality rate was 10.6%, corresponding to an SMR of 2.87 relative to the general population. Men exhibited markedly higher mortality (25.5%) than women did (8.3%), with SMRs of 4.79 and 2.42, respectively, representing nearly a twofold greater risk. Infection was the leading cause of death, followed by cancer and respiratory failure. The excess mortality observed in our cohort was predominantly driven by men, which is consistent with the findings of previous studies [10, 24, 25]. Importantly, evidence from Western and international cohorts suggests that excess mortality in pSD is not uniformly elevated across all patients, but is concentrated within specific high-risk subgroups, particularly men and those with systemic disease involvement [16, 31, 32]. Although a meta-analysis reported that overall SMRs in pSD patients may not be significantly elevated [16], subsequent large-scale studies have consistently demonstrated excess mortality within specific subgroups—particularly men and those with systemic complications [23, 32]. For example, population-based cohorts from Korea and Turkey reported male SMRs of 2.20 and 5.38, respectively—both substantially exceeding those of women [21, 22]. Together with prior international data, our findings therefore reinforce that male sex represents a critical adverse prognostic factor in patients with pSD.

Multivariate analyses revealed that male sex, older age, higher ESSDAI score, ILD, hyponatremia, and elevated levels of CRP and TBIL were independently associated with death. Even after sequential adjustments and forward stepwise modeling, male sex remained a significant prognostic factor, underscoring its robust and independent contribution to death. Stratified analyses further revealed distinct mortality patterns: in men, deaths were predominantly driven by ILD and infection, whereas in women, mortality was more closely linked to systemic inflammation and immune dysregulation—characterized by low C3, elevated CRP, and elevated TBIL. This sex-specific divergence parallels observations from Western cohorts, in which heightened systemic disease activity, pulmonary involvement, and comorbidity burden have been repeatedly linked to increased mortality [17, 32, 33]. Recent prospective and real-world studies have further highlighted pulmonary involvement and infection as major contributors to death, alongside malignancy and cardiovascular disease [31, 33].These results highlight that male sex not only shapes the disease phenotype but also serves as an independent prognostic determinant of mortality in pSD patients. While the direction of these associations is likely biologically generalizable, their clinical expression may be modified by ethnicity- and healthcare-system, related factors, including diagnostic delay, referral patterns, and access to multidisciplinary care [31, 34, 35].

In terms of cancer outcomes, male patients presented a significantly greater incidence of newly developed malignancies than females did (6.4% vs. 2.2%). Kaplan–Meier analysis further confirmed a greater cumulative cancer risk in men. Our analysis revealed that male sex, advanced age, lymphadenopathy, elevated GGT, and hypochloremia were independently associated with cancer. Notably, stepwise Cox analysis for cancer patients revealed that the male HR increased with adjustment, which consistently demonstrated a strong independent association. Furthermore, our analysis revealed that no variables remained independently associated with cancer among man, suggesting that sex differences may play a prominent role in shaping cancer risk in this subgroup. In contrast, among women, longer disease duration, lymphadenopathy, lymphocytopenia, elevated TBIL, and hypochloremia independently increase cancer susceptibility. These findings are broadly consistent with international evidence linking immune dysregulation and B-cell hyperactivity to malignancy risk in pSD, while also suggesting potential sex- and ethnicity-specific oncogenic pathways [31, 35].

In addition, previous studies have established non-Hodgkin lymphoma (NHL) as a major contributor to excess mortality in pSD patients [36–38]. Epidemiological evidence indicates that pSD has the strongest association with NHL among autoimmune diseases, with standardized incidence ratios (SIRs) ranging from 4.6–48.1 compared with those of the general population [19, 39–42]. However, during the follow-up period of our study, only four cases of hematological malignancy and lymphoma were observed (all in women). This likely reflects the limited sample size and relatively short observation period. Taken together, these findings suggest that while the biological links between systemic disease activity, organ involvement, and adverse outcomes may be broadly shared across populations, the dominant clinical pathways leading to mortality and malignancy may differ by ethnicity and healthcare context [16, 31]. Nevertheless, these findings suggest that the spectrum and prognostic impact of malignancies in Chinese patients with pSD may exhibit ethnicity-specific patterns, warranting confirmation in larger cohorts with extended follow-up.

Age- and sex-stratified analyses revealed a cumulative increase in adverse prognostic factors with increasing age, particularly among men. Both sexes demonstrated gradual accumulation of high-risk features, but this trend was more pronounced in males. Men aged ≥ 50 years presented clustering of poor prognostic markers—including middle–high ESSDAI scores, ILD, elevated CRP, elevated TBIL, and hyponatremia—corresponding to markedly higher mortality rates. This age-related risk was substantial; for example, those aged ≥ 75 years had over 30 times the mortality risk of those < 50 years, an effect observed consistently despite no significant interaction with sex. In contrast, women showed a more even distribution of these factors across age groups. Although ILD incidence and disease activity also increased with age, overall mortality remained lower. For cancer risk, a similar but less pronounced pattern was observed. The frequency of lymphadenopathy, hypochloremia, and elevated GGT increased modestly with age, although men presented a higher incidence of new malignancies. Collectively, these findings indicate that aging is associated with widening pre-existing sex disparities in pSD prognosis, with older men exhibiting the highest burden of adverse clinical and biochemical profiles. The underlying mechanisms of these sex-related prognostic differences remain incompletely understood. Immunological and hormonal factors likely play major roles [43]. Women typically exhibit stronger humoral immunity, with estrogens enhancing B-cell activation, antibody production, and Th2/Treg responses [44]. In contrast, androgens favor Th1/IFN-γ pathways and suppress autoantibody generation [45]. These differences may explain why men in our cohort presented lower autoantibody positivity but more severe systemic inflammation and organ involvement at diagnosis. Furthermore, men bore a greater burden of comorbidities—such as ILD, chronic obstructive pulmonary disease, and cardiovascular disease—which compounded their mortality risk. Previous studies also reported that male patients with elevated Ro-52 antibody levels were more prone to pulmonary manifestations [46], further linking sex, immune profiles, and organ-specific outcomes.

This study has several notable strengths. It comprises a large longitudinal cohort of 1,182 patients with pSD, enabling robust characterization of sex-related differences in clinical manifestations and long-term outcomes. The integration of clinical, serological, and survival data, which were combined with multivariable, stepwise Cox regression and interaction analyses, provides strong internal validity. Moreover, SMRs derived from national mortality statistics allowed direct comparisons with population-level expectations, contextualizing the excess mortality risk in pSD. However, several limitations should be acknowledged. First, given the single-center ambispective design, the generalizability of these findings may be limited. Second, potential referral bias and survivor bias stemming from the interval between disease onset and enrollment may exist, particularly among men with aggressive disease. However, this left truncation likely leads to an underestimation of early mortality rather than an overestimation of risk. Thus, the significant excess risk observed in the male cohort represents a conservative and robust estimate. Third, although the median follow-up duration of four years and the limited absolute number of cancer events may have underestimated malignancy incidence, sensitivity analyses confirmed that the direction and magnitude of prognostic associations remained consistent. Future multi-center prospective studies with long term follow-up should be conducted to supplement and validate our findings. Additionally, future studies with access to domain-specific ESSDAI components may further clarify the independent and interactive roles of global disease activity and organ-specific involvement. Despite these limitations, this study offers comprehensive, sex-specific insights into the prognostic heterogeneity of pSD.

### Perspectives and significance

This study shows that male sex is independently associated with higher risks of mortality and malignancy in pSD and acts as a significant effect modifier, rather than merely representing a demographic rarity. The broad implication is that the current, largely sex-neutral approach to pSD risk stratification and management may be insufficient and overlooks a high-risk subgroup. We speculate that the starkly different prognostic pathways, driven by pulmonary and infectious risks in men versus broader systemic inflammation markers in women, reflect distinct, sex-specific disease endotypes. These are likely governed by underlying dichotomies in immune modulation, such as the interplay of sex hormones or X-chromosome-linked mechanisms. Thus, the present study highlights the need to move clinical practice beyond simple risk identification toward elucidating these fundamental biological differences. This work calls for more targeted monitoring protocols and the development of sex-specific therapeutic strategies to address these distinct pathways and improve outcomes for all pSD patients.

## Conclusion

In summary, our findings underscore the importance of sex-specific risk stratification and management in pSD patients. Male patients represent a high-risk subgroup, exhibiting markedly increased risks of mortality and malignancy. Furthermore, the prognostic factors for adverse outcomes differed between the sexes, emphasizing the need for early detection of organ involvement, closer clinical monitoring, and individualized management strategies, which may therefore be critical for improving long-term outcomes, especially in men with pSD.

## Supplementary Information

Additional file 1

Additional file 2

## Data Availability

The data are available upon reasonable request. There are currently no plans to share additional data beyond what is shared in this article.
