# Supplementary material for: Sex differences in primary Sjögren’s disease: prognostic impact on mortality and cancer
Source: Biol Sex Differ. 2026 Jan 14;17:25. doi: 10.1186/s13293-026-00827-7 (PMC12888568; doi:10.1186/s13293-026-00827-7)
Supplement: Supplementary file 1 — Additional file 1 [file 13293_2026_827_MOESM1_ESM.docx]

| **Supplementary Table S1. Sex-specific differences in death and cancer of patients with pSD** | | | | | |
| --- | --- | --- | --- | --- | --- |
| **Variables** | **Causes** | **All (n=1182)** | **Male (n=157)** | **Female (n=1025)** | ***P* value*** |
| **All-cause death** |  | **125 (10.6)** | **40 (25.5)** | **85 (8.3)** | **＜0.001** |
|  | Malignancy | 17 (1.4) | 7 (4.5) | 10 (1.0) | 0.004 |
|  | Infection | 55 (4.7) | 17 (10.8) | 38 (3.7) | ＜0.001 |
|  | Cardiovascular | 11 (0.9) | 2 (1.3) | 9 (0.9) | 0.648 |
|  | Respiratory failure | 14 (1.2) | 5 (3.2) | 9 (0.9) | 0.028 |
|  | Other causes | 16 (1.4) | 4 (2.5) | 12 (1.2) | 0.253 |
|  | Unknown cause | 12 (1.0) | 5 (3.2) | 7 (0.7) | 0.014 |
| **Cancer** |  | **33 (2.8)** | **10 (6.4)** | **23 (2.2)** | **0.008** |
|  | Respiratory system | 10 (0.8) | 7 (4.5) | 3 (0.3) | ＜0.001 |
|  | Digestive system | 7 (0.6) | 2 (1.3) | 5 (0.5) | 0.236 |
|  | Thyroid | 5 (0.4) | 1 (0.6) | 4 (0.4) | 0.510 |
|  | Gynecological/breast | 6 (0.5) | 0 (0.0) | 6 (0.6) | 1.000 |
|  | Tonsil | 1 (0.1) | 0 (0.0) | 1 (0.1) | 1.000 |
|  | Hematological/lymphoid | 4 (0.3) | 0 (0.0) | 4 (0.4) | 1.000 |

Data are presented as the median (IQR) or n (%).

Comparative analyses were conducted between male and female patients with primary Sjögren’s disease.

*Calculated via the *χ²* test or Fisher’s exact test.

ILD: interstitial lung disease. ESSDAI score: European Alliance of Associations for Rheumatology (EULAR) Sjögren’s Syndrome Disease Activity Index score

| **Supplementary Table S2. Univariate and multivariate Cox regression analyses of factors associated with death in patients with pSD** | | | | | | |
| --- | --- | --- | --- | --- | --- | --- |
| **Variables** | **All（n=1182）** | **Survival（n=1057）** | **Death（n=125）** | **Univariate analysis** | **Multivariate analysis** | |
|  |  |  |  | ***P* value*** | **HR (95% CI)** | ***P* value**† |
| **Clinical characteristics** |  |  |  |  |  |  |
| Gender (Male) | 157 (13.3) | 117 (11.1) | 40 (32) | ＜0.001 | 1.998 (1.246,3.203) | 0.004 |
| Age (years) | 58 (49,67) | 58 (48,65) | 69 (63,77) | ＜0.001 | 1.067 (1.045,1.09) | ＜0.001 |
| Disease duration (months) | 36 (9,96) | 36 (12,96) | 24 (3,72) | 0.006 | 0.998 (0.996,1) | 0.113 |
| **Clinical manifestations** |  |  |  |  |  |  |
| Xerostomia | 992 (83.9) | 889 (84.1) | 103 (82.4) | 0.623 | / | / |
| Xerophthalmia | 917 (77.6) | 824 (78) | 93 (74.4) | 0.367 | / | / |
| Fatigue | 564 (47.7) | 498 (47.1) | 66 (52.8) | 0.229 | / | / |
| Fever | 137 (11.6) | 110 (10.4) | 27 (21.6) | ＜0.001 | 1.014 (0.605,1.7) | 0.957 |
| Purpuric rash | 87 (7.4) | 78 (7.4) | 9 (7.2) | 0.940 | / | / |
| Arthralgia | 394 (33.3) | 365 (34.5) | 29 (23.2) | 0.011 | 1.139 (0.7,1.853) | 0.599 |
| Arthritis | 101 (8.5) | 97 (9.2) | 4 (3.2) | 0.024 | 1.445 (0.503,4.156) | 0.494 |
| Dyspnea | 336 (28.4) | 273 (25.8) | 63 (50.4) | ＜0.001 | 1.496 (0.926,2.416) | 0.1 |
| Cough | 394 (33.3) | 325 (30.7) | 69 (55.2) | ＜0.001 | 1.351 (0.83,2.197) | 0.226 |
| Lymphadenopathy | 212 (17.9) | 189 (17.9) | 23 (18.4) | 0.886 | / | / |
| Parotid enlargement | 87 (7.4) | 80 (7.6) | 7 (5.6) | 0.425 | / | / |
| Splenomegaly | 54 (4.6) | 44 (4.2) | 10 (8) | 0.052 | / | / |
| Hemorrhage | 74 (6.3) | 63 (6) | 11 (8.8) | 0.215 | / | / |
| ILD | 372 (31.5) | 299 (28.3) | 73 (58.4) | ＜0.001 | 1.804 (1.163,2.799) | 0.008 |
| ESSDAI score | 7 (3,14) | 6 (3,13) | 14 (6,21) | ＜0.001 | 1.04 (1.013,1.068) | 0.003 |
| **Comorbidities** |  |  |  |  |  |  |
| Hypertension | 302 (25.5) | 253 (23.9) | 49 (39.2) | ＜0.001 | 1.159 (0.756,1.777) | 0.5 |
| Gastroesophageal reflux disease | 284 (24.0) | 249 (23.6) | 35 (28) | 0.272 | / | / |
| Osteoporosis | 273 (23.1) | 236 (22.3) | 37 (29.6) | 0.068 | / | / |
| Dyslipidemia | 217 (18.4) | 197 (18.6) | 20 (16) | 0.471 | / | / |
| Atherosclerosis | 167 (14.1) | 140 (13.2) | 27 (21.6) | 0.011 | 1.177 (0.553,2.505) | 0.671 |
| Hepatic steatosis | 155 (13.1) | 148 (14) | 7 (5.6) | 0.008 | 0.506 (0.225,1.141) | 0.1 |
| Osteoarthritis | 137 (11.6) | 128 (12.1) | 9 (7.2) | 0.105 | / | / |
| Hypothyroidism | 126 (10.7) | 109 (10.3) | 17 (13.6) | 0.260 | / | / |
| Type 2 diabetes mellitus | 108 (9.1) | 85 (8) | 23 (18.4) | ＜0.001 | 1.008 (0.593,1.715) | 0.976 |
| Pulmonary infection | 104 (8.8) | 75 (7.1) | 29 (23.2) | ＜0.001 | 1.443 (0.868,2.4) | 0.157 |
| Ischemic stroke | 104 (8.8) | 81 (7.7) | 23 (18.4) | ＜0.001 | 1.28 (0.755,2.169) | 0.359 |
| Coronary artery disease | 97 (8.2) | 74 (7) | 23 (18.4) | ＜0.001 | 1.135 (0.503,2.559) | 0.761 |
| Liver cyst | 84 (7.1) | 77 (7.3) | 7 (5.6) | 0.488 | / | / |
| Cholelithiasis | 67 (5.7) | 61 (5.8) | 6 (4.8) | 0.657 | / | / |
| Primary biliary cholangitis | 61 (5.2) | 53 (5) | 8 (6.4) | 0.508 | / | / |
| Hyperuricemia | 45 (3.8) | 34 (3.2) | 11 (8.8) | 0.002 | 1.128 (0.559,2.277) | 0.736 |
| Respiratory failure | 42 (3.6) | 27 (2.6) | 15 (12) | ＜0.001 | 1.166 (0.531,2.562) | 0.702 |
| Heart failure | 41 (3.5) | 25 (2.4) | 16 (12.8) | ＜0.001 | 1.629 (0.824,3.221) | 0.161 |
| Chronic obstructive pulmonary disease | 36 (3.0) | 27 (2.6) | 9 (7.2) | 0.004 | 1.239 (0.571,2.687) | 0.587 |
| Cancer | 23 (1.9) | 17 (1.6) | 6 (4.8) | 0.015 | 1.412 (0.564,3.533) | 0.461 |
| **Laboratory features** |  |  |  |  |  |  |
| Leukopenia | 220 (18.6) | 203 (19.2) | 17 (13.6) | 0.128 | / | / |
| Neutropenia | 194 (16.4) | 184 (17.4) | 10 (8) | 0.007 | 0.85 (0.414,1.744) | 0.657 |
| Lymphocytopenia | 300 (25.4) | 257 (24.3) | 43 (34.4) | 0.014 | 1.143 (0.719,1.818) | 0.571 |
| Thrombocytopenia | 174 (14.7) | 146 (13.8) | 28 (22.4) | 0.010 | 1.462 (0.869,2.461) | 0.153 |
| Anemia | 426 (36.0) | 359 (34) | 67 (53.6) | ＜0.001 | 1.059 (0.708,1.584) | 0.781 |
| Elevated ALT | 135 (11.4) | 120 (11.4) | 15 (12) | 0.830 | / | / |
| Elevated AST | 184 (15.6) | 163 (15.4) | 21 (16.8) | 0.688 | / | / |
| Elevated TBIL | 45 (3.8) | 33 (3.1) | 12 (9.6) | 0.002 | 3.46 (1.621,7.386) | 0.001 |
| Elevated GGT | 208 (17.6) | 174 (16.5) | 34 (27.2) | 0.003 | 1.546 (0.961,2.486) | 0.072 |
| Elevated Creatinine | 70 (5.9) | 51 (4.8) | 19 (15.2) | ＜0.001 | 1.042 (0.555,1.956) | 0.898 |
| Hypoalbuminemia | 136 (11.5) | 96 (9.1) | 40 (32) | ＜0.001 | 1.456 (0.877,2.419) | 0.147 |
| Hyponatremia | 137 (11.6) | 101 (9.6) | 36 (28.8) | ＜0.001 | 2.295 (1.36,3.875) | 0.002 |
| Hypokalemia | 103 (8.7) | 93 (8.8) | 10 (8) | 0.765 | / | / |
| Hypochloremia | 30 (2.5) | 18 (1.7) | 12 (9.6) | ＜0.001 | 1.688 (0.788,3.618) | 0.178 |
| Hypergammaglobulinemia | 676 (57.2) | 611 (57.8) | 65 (52) | 0.215 | / | / |
| Hyper-IgG (> 16.2 g/L) | 546 (46.2) | 492 (46.5) | 54 (43.2) | 0.478 | / | / |
| Hyper-IgA (> 3.78 g/L) | 306 (25.9) | 269 (25.4) | 37 (29.6) | 0.316 | / | / |
| Hyper-IgM (> 2.63 g/L) | 88 (7.4) | 77 (7.3) | 11 (8.8) | 0.542 | / | / |
| Hypocomplementemia | 497 (42.0) | 448 (42.4) | 49 (39.2) | 0.495 | / | / |
| Low complement C3 (＜0.70 g/L) | 261 (22.1) | 229 (21.7) | 32 (25.6) | 0.316 | / | / |
| Low complement C4 (＜0.16 g/L) | 409 (34.6) | 369 (34.9) | 40 (32) | 0.518 | / | / |
| Elevated CRP (＞ 8 mg/L) | 296 (25.0) | 227 (21.5) | 69 (55.2) | ＜0.001 | 2.288 (1.487,3.522) | ＜0.001 |
| Elevated ESR (＞ 20 mm/h) | 602 (50.9) | 524 (49.6) | 78 (62.4) | 0.007 | 1.316 (0.827,2.093) | 0.247 |
| ANA titres ≥1:160 | 733 (62.0) | 654 (61.9) | 79 (63.2) | 0.773 | / | / |
| Positive RF | 542 (45.9) | 489 (46.3) | 53 (42.4) | 0.412 | / | / |
| Positive anti-SSA | 843 (71.3) | 766 (72.5) | 77 (61.6) | 0.011 | 1.059 (0.683,1.641) | 0.797 |
| Positive anti-Ro-52 | 679 (57.4) | 614 (58.1) | 65 (52) | 0.193 | / | / |
| Positive anti-SSB | 332 (28.1) | 303 (28.7) | 29 (23.2) | 0.198 | / | / |
| Positive anti-RNP | 84 (7.1) | 80 (7.6) | 4 (3.2) | 0.072 | / | / |
| Positive anti-CENP-B | 103 (8.7) | 89 (8.4) | 14 (11.2) | 0.297 | / | / |
| Positive anti-AMA-M2 | 103 (8.7) | 96 (9.1) | 7 (5.6) | 0.192 | / | / |

Data are presented as median (IQR) or n (%). Comparative analyses were conducted between with and without cancer groups of patients with primary Sjögren’s disease.

* Calculated using the *χ²* test or Fisher’s exact test for categorical variables, and the Mann-Whitney U test for continuous variables.

† Variables with *P* < 0.05 in the univariate analysis were included in the multivariable Cox regression model.

ILD: interstitial lung disease. ESSDAI score: European Alliance of Associations for Rheumatology (EULAR) Sjögren’s Syndrome Disease Activity Index score. ALT: alanine aminotransferase. AST: aspartate aminotransferase. TBIL: total bilirubin. GGT: γ-glutamyl transferase. Hyper-IgG: Hypergammaglobulinemia with elevated immunoglobulin G. Hyper-IgA: Hypergammaglobulinemia with elevated immunoglobulin A. Hyper-IgM: Hypergammaglobulinemia with elevated immunoglobulin M. Elevated CRP: elevated C-reactive protein. Elevated ESR: elevated erythrocyte sedimentation rate. HR: hazard ratio. CI: confidence intervals.

| **Supplementary Table S3. Fully adjusted Cox regression analysis of factors associated with death in patients with pSD** | | | | | |
| --- | --- | --- | --- | --- | --- |
| **Variables** | **All**（n=1182） | **Survival（n=1057）** | **Death（n=125）** | **Multivariate analysis** | |
|  |  |  |  | **HR (95% CI)** | **P value†** |
| **Clinical characteristics** |  |  |  |  |  |
| Gender (Male) | 157 (13.3) | 117 (11.1) | 40 (32) | 2.451 (1.419, 4.235) | 0.001 |
| Age (years) | 58 (49,67) | 58 (48,65) | 69 (63,77) | 1.07 (1.045, 1.096) | ＜0.001 |
| Disease duration (months) | 36 (9,96) | 36 (12,96) | 24 (3,72) | 0.998 (0.995, 1.001) | 0.129 |
| **Clinical manifestations** |  |  |  |  |  |
| Xerostomia | 992 (83.9) | 889 (84.1) | 103 (82.4) | 2.171 (1.171, 4.023) | 0.014 |
| Xerophthalmia | 917 (77.6) | 824 (78) | 93 (74.4) | 0.804 (0.475, 1.359) | 0.415 |
| Fatigue | 564 (47.7) | 498 (47.1) | 66 (52.8) | 0.858 (0.549, 1.341) | 0.502 |
| Fever | 137 (11.6) | 110 (10.4) | 27 (21.6) | 0.685 (0.367, 1.278) | 0.234 |
| Purpuric rash | 87 (7.4) | 78 (7.4) | 9 (7.2) | 0.508 (0.207, 1.246) | 0.139 |
| Arthralgia | 394 (33.3) | 365 (34.5) | 29 (23.2) | 0.787 (0.456, 1.358) | 0.39 |
| Arthritis | 101 (8.5) | 97 (9.2) | 4 (3.2) | 0.727 (0.236, 2.243) | 0.58 |
| Dyspnea | 336 (28.4) | 273 (25.8) | 63 (50.4) | 1.84 (1.103, 3.07) | 0.02 |
| Cough | 394 (33.3) | 325 (30.7) | 69 (55.2) | 1.35 (0.81, 2.253) | 0.25 |
| Lymphadenopathy | 212 (17.9) | 189 (17.9) | 23 (18.4) | 0.857 (0.463, 1.587) | 0.623 |
| Parotid enlargement | 87 (7.4) | 80 (7.6) | 7 (5.6) | 0.48 (0.183, 1.259) | 0.136 |
| Splenomegaly | 54 (4.6) | 44 (4.2) | 10 (8) | 0.856 (0.352, 2.085) | 0.732 |
| Hemorrhage | 74 (6.3) | 63 (6) | 11 (8.8) | 1.84 (0.768, 4.405) | 0.171 |
| ILD | 372 (31.5) | 299 (28.3) | 73 (58.4) | 1.975 (1.211, 3.221) | 0.006 |
| ESSDAI score | 7 (3,14) | 6 (3,13) | 14 (6,21) | 1.053 (1.02, 1.088) | 0.001 |
| **Comorbidities** |  |  |  |  |  |
| Hypertension | 302 (25.5) | 253 (23.9) | 49 (39.2) | 0.799 (0.503, 1.269) | 0.342 |
| Gastroesophageal reflux disease | 284 (24.0) | 249 (23.6) | 35 (28) | 0.588 (0.351, 0.986) | 0.044 |
| Osteoporosis | 273 (23.1) | 236 (22.3) | 37 (29.6) | 1.629 (1.001, 2.65) | 0.049 |
| Dyslipidemia | 217 (18.4) | 197 (18.6) | 20 (16) | 0.781 (0.433, 1.408) | 0.411 |
| Atherosclerosis | 167 (14.1) | 140 (13.2) | 27 (21.6) | 1.268 (0.564, 2.849) | 0.566 |
| Hepatic steatosis | 155 (13.1) | 148 (14) | 7 (5.6) | 0.589 (0.25, 1.389) | 0.227 |
| Osteoarthritis | 137 (11.6) | 128 (12.1) | 9 (7.2) | 0.602 (0.261, 1.392) | 0.236 |
| Hypothyroidism | 126 (10.7) | 109 (10.3) | 17 (13.6) | 1.132 (0.588, 2.181) | 0.71 |
| Type 2 diabetes mellitus | 108 (9.1) | 85 (8) | 23 (18.4) | 1.028 (0.568, 1.861) | 0.927 |
| Pulmonary infection | 104 (8.8) | 75 (7.1) | 29 (23.2) | 1.546 (0.866, 2.759) | 0.14 |
| Ischemic stroke | 104 (8.8) | 81 (7.7) | 23 (18.4) | 1.444 (0.814, 2.562) | 0.209 |
| Coronary artery disease | 97 (8.2) | 74 (7) | 23 (18.4) | 0.877 (0.368, 2.092) | 0.767 |
| Liver cyst | 84 (7.1) | 77 (7.3) | 7 (5.6) | 0.799 (0.336, 1.901) | 0.612 |
| Cholelithiasis | 67 (5.7) | 61 (5.8) | 6 (4.8) | 0.564 (0.218, 1.46) | 0.238 |
| Primary biliary cholangitis | 61 (5.2) | 53 (5) | 8 (6.4) | 4.109 (1.468, 11.505) | 0.007 |
| Hyperuricemia | 45 (3.8) | 34 (3.2) | 11 (8.8) | 1.345 (0.6, 3.015) | 0.471 |
| Respiratory failure | 42 (3.6) | 27 (2.6) | 15 (12) | 1.283 (0.558, 2.948) | 0.558 |
| Heart failure | 41 (3.5) | 25 (2.4) | 16 (12.8) | 1.528 (0.722, 3.235) | 0.268 |
| Chronic obstructive pulmonary disease | 36 (3.0) | 27 (2.6) | 9 (7.2) | 1.182 (0.504, 2.776) | 0.701 |
| Cancer | 23 (1.9) | 17 (1.6) | 6 (4.8) | 1.697 (0.606, 4.752) | 0.314 |
| **Laboratory features** |  |  |  |  |  |
| Leukopenia | 220 (18.6) | 203 (19.2) | 17 (13.6) | 0.975 (0.395, 2.406) | 0.956 |
| Neutropenia | 194 (16.4) | 184 (17.4) | 10 (8) | 0.815 (0.296, 2.242) | 0.692 |
| Lymphocytopenia | 300 (25.4) | 257 (24.3) | 43 (34.4) | 0.911 (0.543, 1.526) | 0.722 |
| Thrombocytopenia | 174 (14.7) | 146 (13.8) | 28 (22.4) | 1.27 (0.677, 2.384) | 0.456 |
| Anemia | 426 (36.0) | 359 (34) | 67 (53.6) | 1.031 (0.656, 1.62) | 0.894 |
| Elevated ALT | 135 (11.4) | 120 (11.4) | 15 (12) | 2.313 (1.067, 5.012) | 0.034 |
| Elevated AST | 184 (15.6) | 163 (15.4) | 21 (16.8) | 0.387 (0.185, 0.809) | 0.012 |
| Elevated TBIL | 45 (3.8) | 33 (3.1) | 12 (9.6) | 3.732 (1.621, 8.594) | 0.002 |
| Elevated GGT | 208 (17.6) | 174 (16.5) | 34 (27.2) | 1.71 (0.972, 3.007) | 0.063 |
| Elevated Creatinine | 70 (5.9) | 51 (4.8) | 19 (15.2) | 0.848 (0.403, 1.784) | 0.664 |
| Hypoalbuminemia | 136 (11.5) | 96 (9.1) | 40 (32) | 0.801 (0.449, 1.427) | 0.451 |
| Hyponatremia | 137 (11.6) | 101 (9.6) | 36 (28.8) | 2.536 (1.411, 4.56) | 0.002 |
| Hypokalemia | 103 (8.7) | 93 (8.8) | 10 (8) | 0.931 (0.436, 1.988) | 0.854 |
| Hypochloremia | 30 (2.5) | 18 (1.7) | 12 (9.6) | 2.234 (0.973, 5.128) | 0.058 |
| Hyper-IgG (> 16.2 g/L) | 546 (46.2) | 492 (46.5) | 54 (43.2) | 1.055 (0.64, 1.74) | 0.833 |
| Hyper-IgA (> 3.78 g/L) | 306 (25.9) | 269 (25.4) | 37 (29.6) | 0.963 (0.596, 1.555) | 0.876 |
| Hyper-IgM (> 2.63 g/L) | 88 (7.4) | 77 (7.3) | 11 (8.8) | 0.988 (0.452, 2.157) | 0.975 |
| Low complement C3 (＜0.70 g/L) | 261 (22.1) | 229 (21.7) | 32 (25.6) | 1.708 (0.954, 3.059) | 0.072 |
| Low complement C4 (＜0.16 g/L) | 409 (34.6) | 369 (34.9) | 40 (32) | 0.973 (0.565, 1.677) | 0.922 |
| Elevated CRP (＞ 8 mg/L) | 296 (25.0) | 227 (21.5) | 69 (55.2) | 3.441 (2.122, 5.581) | ＜0.001 |
| Elevated ESR (＞ 20 mm/h) | 602 (50.9) | 524 (49.6) | 78 (62.4) | 0.749 (0.447, 1.254) | 0.272 |
| ANA titres ≥1:160 | 733 (62.0) | 654 (61.9) | 79 (63.2) | 0.832 (0.489, 1.416) | 0.497 |
| Positive RF | 542 (45.9) | 489 (46.3) | 53 (42.4) | 1.176 (0.706, 1.959) | 0.534 |
| Positive anti-SSA | 843 (71.3) | 766 (72.5) | 77 (61.6) | 1.128 (0.663, 1.918) | 0.656 |
| Positive anti-Ro-52 | 679 (57.4) | 614 (58.1) | 65 (52) | 1.341 (0.837, 2.15) | 0.222 |
| Positive anti-SSB | 332 (28.1) | 303 (28.7) | 29 (23.2) | 0.748 (0.417, 1.342) | 0.331 |
| Positive anti-RNP | 84 (7.1) | 80 (7.6) | 4 (3.2) | 0.652 (0.216, 1.971) | 0.449 |
| Positive anti-CENP-B | 103 (8.7) | 89 (8.4) | 14 (11.2) | 1.373 (0.627, 3.007) | 0.428 |
| Positive anti-AMA-M2 | 103 (8.7) | 96 (9.1) | 7 (5.6) | 0.264 (0.104, 0.67) | 0.005 |

Data are presented as median (IQR) or n (%). Comparative analyses were conducted between with and without cancer groups of patients with primary Sjögren’s disease.

† Calculated using the multivariable Cox regression model.

ILD: interstitial lung disease. ESSDAI score: European Alliance of Associations for Rheumatology (EULAR) Sjögren’s Syndrome Disease Activity Index score. ALT: alanine aminotransferase. AST: aspartate aminotransferase. TBIL: total bilirubin. GGT: γ-glutamyl transferase. Hyper-IgG: Hypergammaglobulinemia with elevated immunoglobulin G. Hyper-IgA: Hypergammaglobulinemia with elevated immunoglobulin A. Hyper-IgM: Hypergammaglobulinemia with elevated immunoglobulin M. Elevated CRP: elevated C-reactive protein. Elevated ESR: elevated erythrocyte sedimentation rate. HR: hazard ratio. CI: confidence intervals.

| **Supplementary Table S4. Univariate and multivariate Cox regression analyses of factors associated with cancer in patients with pSD** | | | | | | |
| --- | --- | --- | --- | --- | --- | --- |
| **Variables** | **All (n=1159)** | **Without cancer (n=1126)** | **With cancer (n=33)** | **Univariate analysis** | **Multivariate analysis** | |
|  |  |  |  | **P value*** | **HR (95% CI)** | ***P* value†** |
| **Clinical characteristics** |  |  |  |  |  |  |
| Gender (Male) | 152 (13.1) | 142 (12.6) | 10 (30.3) | 0.007 | 3.799 (1.754,8.231) | 0.001 |
| Age (years) | 58 (49,67) | 58 (49,66) | 64 (54.25,72.75) | 0.037 | 1.038 (1.008,1.069) | 0.013 |
| Disease duration (months) | 36 (9,96) | 36 (9,96) | 60 (24,120) | 0.227 | / | / |
| **Clinical manifestations** |  |  |  |  | / | / |
| Xerostomia | 977 (84.3) | 949 (84.3) | 28 (84.8) | 0.930 | / | / |
| Xerophthalmia | 901 (77.7) | 874 (77.6) | 27 (81.8) | 0.568 | / | / |
| Fatigue | 556 (48) | 540 (48) | 16 (48.5) | 0.952 | / | / |
| Fever | 133 (11.5) | 127 (11.3) | 6 (18.2) | 0.260 | / | / |
| Purpuric rash | 86 (7.4) | 83 (7.4) | 3 (9.1) | 0.731 | / | / |
| Arthralgia | 391 (33.7) | 380 (33.7) | 11 (33.3) | 0.960 | / | / |
| Arthritis | 101 (8.7) | 96 (8.5) | 5 (15.2) | 0.200 | / | / |
| Dyspnea | 326 (28.1) | 319 (28.3) | 7 (21.2) | 0.370 | / | / |
| Cough | 385 (33.2) | 375 (33.3) | 10 (30.3) | 0.718 | / | / |
| Lymphadenopathy | 207 (17.9) | 196 (17.4) | 11 (33.3) | 0.019 | 2.495 (1.178,5.285) | 0.017 |
| Parotid enlargement | 86 (7.4) | 83 (7.4) | 3 (9.1) | 0.731 | / | / |
| Splenomegaly | 51 (4.4) | 48 (4.3) | 3 (9.1) | 0.174 | / | / |
| Hemorrhage | 72 (6.2) | 68 (6) | 4 (12.1) | 0.143 | / | / |
| ILD | 360 (31.1) | 348 (30.9) | 12 (36.4) | 0.504 | / | / |
| ESSDAI score | 7 (3,13) | 7 (3,13) | 7.5 (4,16.5) | 0.306 | / | / |
| **Comorbidities** |  |  |  |  | / | / |
| Hypertension | 299 (25.8) | 288 (25.6) | 11 (33.3) | 0.316 | / | / |
| Gastroesophageal reflux disease | 279 (24.1) | 268 (23.8) | 11 (33.3) | 0.207 | / | / |
| Osteoporosis | 270 (23.3) | 261 (23.2) | 9 (27.3) | 0.583 | / | / |
| Dyslipidemia | 213 (18.4) | 211 (18.7) | 2 (6.1) | 0.064 | / | / |
| Atherosclerosis | 164 (14.2) | 159 (14.1) | 5 (15.2) | 0.801 | / | / |
| Hepatic steatosis | 155 (13.4) | 148 (13.1) | 7 (21.2) | 0.191 | / | / |
| Osteoarthritis | 136 (11.7) | 131 (11.6) | 5 (15.2) | 0.579 | / | / |
| Hypothyroidism | 125 (10.8) | 120 (10.7) | 5 (15.2) | 0.391 | / | / |
| Type 2 diabetes mellitus | 107 (9.2) | 102 (9.1) | 5 (15.2) | 0.221 | / | / |
| Pulmonary infection | 103 (8.9) | 97 (8.6) | 6 (18.2) | 0.065 | / | / |
| Ischemic stroke | 101 (8.7) | 97 (8.6) | 4 (12.1) | 0.523 | / | / |
| Coronary artery disease | 95 (8.2) | 93 (8.3) | 2 (6.1) | 1.000 | / | / |
| Liver cyst | 82 (7.1) | 76 (6.7) | 6 (18.2) | 0.025 | 2.355 (0.939,5.902) | 0.068 |
| Cholelithiasis | 64 (5.5) | 60 (5.3) | 4 (12.1) | 0.104 | / | / |
| Primary biliary cholangitis | 60 (5.2) | 56 (5) | 4 (12.1) | 0.086 | / | / |
| Hyperuricemia | 45 (3.9) | 45 (4) | 0 (0) | 0.635 | / | / |
| Respiratory failure | 42 (3.6) | 41 (3.6) | 1 (3) | 1.000 | / | / |
| Heart failure | 39 (3.4) | 38 (3.4) | 1 (3) | 1.000 | / | / |
| Chronic obstructive pulmonary disease | 36 (3.1) | 35 (3.1) | 1 (3) | 1.000 | / | / |
| **Laboratory features** |  |  |  |  |  |  |
| Leukopenia | 214 (18.5) | 207 (18.4) | 7 (21.2) | 0.680 | / | / |
| Neutropenia | 190 (16.4) | 185 (16.4) | 5 (15.2) | 0.845 | / | / |
| Lymphocytopenia | 294 (25.4) | 280 (24.9) | 14 (42.4) | 0.022 | 1.704 (0.837,3.472) | 0.142 |
| Thrombocytopenia | 167 (14.4) | 162 (14.4) | 5 (15.2) | 0.804 | / | / |
| Anemia | 414 (35.7) | 396 (35.2) | 18 (54.5) | 0.022 | 1.487 (0.732,3.018) | 0.273 |
| Elevated ALT | 131 (11.3) | 127 (11.3) | 4 (12.1) | 0.782 | / | / |
| Elevated AST | 178 (15.4) | 172 (15.3) | 6 (18.2) | 0.628 | / | / |
| Elevated TBIL | 42 (3.6) | 39 (3.5) | 3 (9.1) | 0.088 | / | / |
| Elevated GGT | 203 (17.5) | 192 (17.1) | 11 (33.3) | 0.015 | 2.444 (1.167,5.119) | 0.018 |
| Elevated Creatinine | 67 (5.8) | 67 (6) | 0 (0) | 0.254 | / | / |
| Hypoalbuminemia | 132 (11.4) | 128 (11.4) | 4 (12.1) | 0.784 | / | / |
| Hyponatremia | 134 (11.6) | 128 (11.4) | 6 (18.2) | 0.262 | / | / |
| Hypokalemia | 102 (8.8) | 101 (9) | 1 (3) | 0.354 | / | / |
| Hypochloremia | 29 (2.5) | 25 (2.2) | 4 (12.1) | 0.008 | 3.494 (1.143,10.68) | 0.028 |
| Hypergammaglobulinemia | 668 (57.6) | 650 (57.7) | 18 (54.5) | 0.715 | / | / |
| Hyper-IgG (> 16.2 g/L) | 540 (46.6) | 526 (46.7) | 14 (42.4) | 0.626 | / | / |
| Hyper-IgA (> 3.78 g/L) | 303 (26.1) | 291 (25.8) | 12 (36.4) | 0.175 | / | / |
| Hyper-IgM (> 2.63 g/L) | 86 (7.4) | 83 (7.4) | 3 (9.1) | 0.731 | / | / |
| Hypocomplementemia | 489 (42.2) | 476 (42.3) | 13 (39.4) | 0.741 | / | / |
| Low complement C3 (＜0.70 g/L) | 254 (21.9) | 249 (22.1) | 5 (15.2) | 0.341 | / | / |
| Low complement C4 (＜0.16 g/L) | 404 (34.9) | 392 (34.8) | 12 (36.4) | 0.854 | / | / |
| Elevated CRP (＞ 8 mg/L) | 284 (24.5) | 273 (24.2) | 11 (33.3) | 0.232 | / | / |
| Elevated ESR (＞ 20 mm/h) | 589 (50.8) | 572 (50.8) | 17 (51.5) | 0.935 | / | / |
| ANA titres ≥1:160 | 721 (62.2) | 699 (62.1) | 22 (66.7) | 0.592 | / | / |
| Positive RF | 534 (46.1) | 521 (46.3) | 13 (39.4) | 0.435 | / | / |
| Positive anti-SSA | 827 (71.4) | 805 (71.5) | 22 (66.7) | 0.546 | / | / |
| Positive anti-Ro-52 | 665 (57.4) | 645 (57.3) | 20 (60.6) | 0.704 | / | / |
| Positive anti-SSB | 327 (28.2) | 316 (28.1) | 11 (33.3) | 0.507 | / | / |
| Positive anti-RNP | 82 (7.1) | 77 (6.8) | 5 (15.2) | 0.078 | / | / |
| Positive anti-CENP-B | 100 (8.6) | 94 (8.3) | 6 (18.2) | 0.058 | / | / |
| Positive anti-AMA-M2 | 102 (8.8) | 97 (8.6) | 5 (15.2) | 0.203 | / | / |

Data are presented as median (IQR) or n (%). Comparative analyses were conducted between with and without cancer groups of patients with primary Sjögren’s disease.

* Calculated using the *χ²* test or Fisher’s exact test for categorical variables, and the Mann-Whitney U test for continuous variables.

† Variables with *P* < 0.05 in the univariate analysis were included in the multivariable Cox regression model.

ILD: interstitial lung disease. ESSDAI score: European Alliance of Associations for Rheumatology (EULAR) Sjögren’s Syndrome Disease Activity Index score. ALT: alanine aminotransferase. AST: aspartate aminotransferase. TBIL: total bilirubin. GGT: γ-glutamyl transferase. Hyper-IgG: Hypergammaglobulinemia with elevated immunoglobulin G. Hyper-IgA: Hypergammaglobulinemia with elevated immunoglobulin A. Hyper-IgM: Hypergammaglobulinemia with elevated immunoglobulin M. Elevated CRP: elevated C-reactive protein. Elevated ESR: elevated erythrocyte sedimentation rate. HR: hazard ratio. CI: confidence intervals.

| **Supplementary Table S5. Fully adjusted Cox regression analysis of factors associated with cancer in patients with pSD** | | | | | |
| --- | --- | --- | --- | --- | --- |
| **Variables** | **All (n=1159)** | **Without cancer (n=1126)** | **With cancer (n=33)** | **Multivariate analysis** | |
|  |  |  |  | **HR (95% CI)** | **P value†** |
| **Clinical characteristics** |  |  |  |  |  |
| Gender (Male) | 157 (13.3) | 117 (11.1) | 40 (32) | 10.154 (3.041, 33.902) | ＜0.001 |
| Age (years) | 58 (49,67) | 58 (48,65) | 69 (63,77) | 1.046 (1.004, 1.09) | 0.03 |
| Disease duration (months) | 36 (9,96) | 36 (12,96) | 24 (3,72) | 1.004 (1, 1.008) | 0.037 |
| **Clinical manifestations** |  |  |  |  |  |
| Xerostomia | 992 (83.9) | 889 (84.1) | 103 (82.4) | 1.229 (0.376, 4.018) | 0.733 |
| Xerophthalmia | 917 (77.6) | 824 (78) | 93 (74.4) | 0.927 (0.311, 2.765) | 0.892 |
| Fatigue | 564 (47.7) | 498 (47.1) | 66 (52.8) | 1.365 (0.561, 3.32) | 0.493 |
| Fever | 137 (11.6) | 110 (10.4) | 27 (21.6) | 0.605 (0.168, 2.177) | 0.441 |
| Purpuric rash | 87 (7.4) | 78 (7.4) | 9 (7.2) | 1.083 (0.201, 5.845) | 0.926 |
| Arthralgia | 394 (33.3) | 365 (34.5) | 29 (23.2) | 0.653 (0.212, 2.01) | 0.457 |
| Arthritis | 101 (8.5) | 97 (9.2) | 4 (3.2) | 2.162 (0.511, 9.142) | 0.295 |
| Dyspnea | 336 (28.4) | 273 (25.8) | 63 (50.4) | 0.461 (0.125, 1.706) | 0.246 |
| Cough | 394 (33.3) | 325 (30.7) | 69 (55.2) | 0.889 (0.299, 2.642) | 0.832 |
| Lymphadenopathy | 212 (17.9) | 189 (17.9) | 23 (18.4) | 5.291 (1.85, 15.139) | 0.002 |
| Parotid enlargement | 87 (7.4) | 80 (7.6) | 7 (5.6) | 0.868 (0.192, 3.914) | 0.854 |
| Splenomegaly | 54 (4.6) | 44 (4.2) | 10 (8) | 1.104 (0.165, 7.374) | 0.919 |
| Hemorrhage | 74 (6.3) | 63 (6) | 11 (8.8) | 2.923 (0.63, 13.565) | 0.171 |
| ILD | 372 (31.5) | 299 (28.3) | 73 (58.4) | 0.867 (0.334, 2.248) | 0.768 |
| ESSDAI score | 7 (3,14) | 6 (3,13) | 14 (6,21) | 0.956 (0.882, 1.036) | 0.273 |
| **Comorbidities** |  |  |  |  |  |
| Hypertension | 302 (25.5) | 253 (23.9) | 49 (39.2) | 1.002 (0.347, 2.896) | 0.997 |
| Gastroesophageal reflux disease | 284 (24.0) | 249 (23.6) | 35 (28) | 2.314 (0.833, 6.425) | 0.107 |
| Osteoporosis | 273 (23.1) | 236 (22.3) | 37 (29.6) | 1.294 (0.447, 3.75) | 0.634 |
| Dyslipidemia | 217 (18.4) | 197 (18.6) | 20 (16) | 0.195 (0.038, 0.997) | 0.05 |
| Atherosclerosis | 167 (14.1) | 140 (13.2) | 27 (21.6) | 0.664 (0.141, 3.124) | 0.605 |
| Hepatic steatosis | 155 (13.1) | 148 (14) | 7 (5.6) | 2.243 (0.751, 6.694) | 0.148 |
| Osteoarthritis | 137 (11.6) | 128 (12.1) | 9 (7.2) | 0.929 (0.228, 3.785) | 0.918 |
| Hypothyroidism | 126 (10.7) | 109 (10.3) | 17 (13.6) | 2.803 (0.828, 9.493) | 0.098 |
| Type 2 diabetes mellitus | 108 (9.1) | 85 (8) | 23 (18.4) | 1.424 (0.366, 5.535) | 0.61 |
| Pulmonary infection | 104 (8.8) | 75 (7.1) | 29 (23.2) | 2.448 (0.673, 8.898) | 0.174 |
| Ischemic stroke | 104 (8.8) | 81 (7.7) | 23 (18.4) | 0.912 (0.221, 3.764) | 0.899 |
| Coronary artery disease | 97 (8.2) | 74 (7) | 23 (18.4) | 1.055 (0.121, 9.165) | 0.962 |
| Liver cyst | 84 (7.1) | 77 (7.3) | 7 (5.6) | 2.042 (0.58, 7.185) | 0.266 |
| Cholelithiasis | 67 (5.7) | 61 (5.8) | 6 (4.8) | 2.064 (0.529, 8.047) | 0.297 |
| Primary biliary cholangitis | 61 (5.2) | 53 (5) | 8 (6.4) | 1.544 (0.27, 8.812) | 0.625 |
| Hyperuricemia | 45 (3.8) | 34 (3.2) | 11 (8.8) | 0 (0,0) | 0.99 |
| Respiratory failure | 42 (3.6) | 27 (2.6) | 15 (12) | 0.289 (0.019, 4.408) | 0.372 |
| Heart failure | 41 (3.5) | 25 (2.4) | 16 (12.8) | 4.252 (0.268, 67.42) | 0.305 |
| Chronic obstructive pulmonary disease | 36 (3.0) | 27 (2.6) | 9 (7.2) | 0.855 (0.085, 8.614) | 0.895 |
| **Laboratory features** |  |  |  |  |  |
| Leukopenia | 220 (18.6) | 203 (19.2) | 17 (13.6) | 0.597 (0.11, 3.23) | 0.549 |
| Neutropenia | 194 (16.4) | 184 (17.4) | 10 (8) | 0.723 (0.131, 4) | 0.71 |
| Lymphocytopenia | 300 (25.4) | 257 (24.3) | 43 (34.4) | 3.171 (1.027, 9.79) | 0.045 |
| Thrombocytopenia | 174 (14.7) | 146 (13.8) | 28 (22.4) | 0.516 (0.126, 2.111) | 0.357 |
| Anemia | 426 (36.0) | 359 (34) | 67 (53.6) | 2.045 (0.856, 4.889) | 0.108 |
| Elevated ALT | 135 (11.4) | 120 (11.4) | 15 (12) | 0.914 (0.177, 4.714) | 0.915 |
| Elevated AST | 184 (15.6) | 163 (15.4) | 21 (16.8) | 0.848 (0.168, 4.272) | 0.841 |
| Elevated TBIL | 45 (3.8) | 33 (3.1) | 12 (9.6) | 2.664 (0.505, 14.061) | 0.248 |
| Elevated GGT | 208 (17.6) | 174 (16.5) | 34 (27.2) | 2.164 (0.68, 6.889) | 0.191 |
| Elevated Creatinine | 70 (5.9) | 51 (4.8) | 19 (15.2) | 0 (0, .) | 0.981 |
| Hypoalbuminemia | 136 (11.5) | 96 (9.1) | 40 (32) | 1.657 (0.37, 7.416) | 0.509 |
| Hyponatremia | 137 (11.6) | 101 (9.6) | 36 (28.8) | 1.004 (0.214, 4.702) | 0.996 |
| Hypokalemia | 103 (8.7) | 93 (8.8) | 10 (8) | 0.085 (0.007, 1.032) | 0.053 |
| Hypochloremia | 30 (2.5) | 18 (1.7) | 12 (9.6) | 6.109 (0.947, 39.391) | 0.057 |
| Hyper-IgG (> 16.2 g/L) | 546 (46.2) | 492 (46.5) | 54 (43.2) | 0.847 (0.323, 2.221) | 0.736 |
| Hyper-IgA (> 3.78 g/L) | 306 (25.9) | 269 (25.4) | 37 (29.6) | 1.402 (0.542, 3.627) | 0.485 |
| Hyper-IgM (> 2.63 g/L) | 88 (7.4) | 77 (7.3) | 11 (8.8) | 0.643 (0.111, 3.736) | 0.623 |
| Low complement C3 (＜0.70 g/L) | 261 (22.1) | 229 (21.7) | 32 (25.6) | 0.382 (0.104, 1.41) | 0.149 |
| Low complement C4 (＜0.16 g/L) | 409 (34.6) | 369 (34.9) | 40 (32) | 1.462 (0.569, 3.756) | 0.43 |
| Elevated CRP (＞ 8 mg/L) | 296 (25.0) | 227 (21.5) | 69 (55.2) | 0.798 (0.269, 2.366) | 0.685 |
| Elevated ESR (＞ 20 mm/h) | 602 (50.9) | 524 (49.6) | 78 (62.4) | 0.585 (0.211, 1.623) | 0.303 |
| ANA titres ≥1:160 | 733 (62.0) | 654 (61.9) | 79 (63.2) | 0.701 (0.226, 2.17) | 0.538 |
| Positive RF | 542 (45.9) | 489 (46.3) | 53 (42.4) | 1.078 (0.362, 3.204) | 0.893 |
| Positive anti-SSA | 843 (71.3) | 766 (72.5) | 77 (61.6) | 0.788 (0.264, 2.349) | 0.669 |
| Positive anti-Ro-52 | 679 (57.4) | 614 (58.1) | 65 (52) | 1.879 (0.642, 5.504) | 0.25 |
| Positive anti-SSB | 332 (28.1) | 303 (28.7) | 29 (23.2) | 1.303 (0.437, 3.884) | 0.635 |
| Positive anti-RNP | 84 (7.1) | 80 (7.6) | 4 (3.2) | 4.15 (1.143, 15.069) | 0.031 |
| Positive anti-CENP-B | 103 (8.7) | 89 (8.4) | 14 (11.2) | 2.911 (0.787, 10.769) | 0.109 |
| Positive anti-AMA-M2 | 103 (8.7) | 96 (9.1) | 7 (5.6) | 2.46 (0.627, 9.658) | 0.197 |

Data are presented as median (IQR) or n (%). Comparative analyses were conducted between with and without cancer groups of patients with primary Sjögren’s disease.

† Calculated using the multivariable Cox regression model.

ILD: interstitial lung disease. ESSDAI score: European Alliance of Associations for Rheumatology (EULAR) Sjögren’s Syndrome Disease Activity Index score. ALT: alanine aminotransferase. AST: aspartate aminotransferase. TBIL: total bilirubin. GGT: γ-glutamyl transferase. Hyper-IgG: Hypergammaglobulinemia with elevated immunoglobulin G. Hyper-IgA: Hypergammaglobulinemia with elevated immunoglobulin A. Hyper-IgM: Hypergammaglobulinemia with elevated immunoglobulin M. Elevated CRP: elevated C-reactive protein. Elevated ESR: elevated erythrocyte sedimentation rate. HR: hazard ratio. CI: confidence intervals.

| **Supplementary Table S6. Univariate and multivariate Cox regression analyses of factors associated with death in male patients with pSD** | | | | | | |
| --- | --- | --- | --- | --- | --- | --- |
| **Variables** | **All (n=157)** | **Survival (n=117)** | **Death (n=40)** | **Univariate analysis** | **Multivariate analysis** | |
|  |  |  |  | **P value*** | **HR (95%CI)** | **P value†** |
| **Clinical characteristics** |  |  |  |  |  |  |
| Age (years) | 64 (54,70) | 61 (50,68) | 64.25 (68,73) | ＜0.001 | 1.043 (0.995,1.092) | 0.078 |
| Disease duration (months) | 12 (3,48) | 12 (3,48) | 4.5 (1,36) | 0.034 | 1.001 (0.991,1.011) | 0.835 |
| **Clinical manifestations** |  |  |  |  |  |  |
| Xerostomia | 109 (69.4) | 79 (67.5) | 30 (75) | 0.375 | / | / |
| Xerophthalmia | 102 (65) | 76 (65) | 26 (65) | 0.996 | / | / |
| Fatigue | 59 (37.6) | 40 (34.2) | 19 (47.5) | 0.133 | / | / |
| Fever | 25 (15.9) | 16 (13.7) | 9 (22.5) | 0.188 | / | / |
| Purpuric rash | 7 (4.5) | 6 (5.1) | 1 (2.5) | 0.679 | / | / |
| Arthralgia | 29 (18.5) | 21 (17.9) | 8 (20) | 0.773 | / | / |
| Arthritis | 8 (5.1) | 7 (6) | 1 (2.5) | 0.681 | / | / |
| Dyspnea | 74 (47.1) | 52 (44.4) | 22 (55) | 0.248 | / | / |
| Cough | 86 (54.8) | 59 (50.4) | 27 (67.5) | 0.061 | / | / |
| Lymphadenopathy | 27 (17.2) | 20 (17.1) | 7 (17.5) | 0.953 | / | / |
| Parotid enlargement | 11 (7) | 8 (6.8) | 3 (7.5) | 1.000 | / | / |
| Splenomegaly | 6 (3.8) | 4 (3.4) | 2 (5) | 0.645 | / | / |
| Hemorrhage | 8 (5.1) | 7 (6) | 1 (2.5) | 0.681 | / | / |
| ILD | 90 (57.3) | 56 (47.9) | 34 (85) | ＜0.001 | 7.35 (2.15,25.121) | 0.001 |
| ESSDAI score | 10 (3,16.5) | 6 (2,15) | 16.55±1.66 | ＜0.001 | 1.007 (0.958,1.059) | 0.78 |
| **Comorbidities** |  |  |  |  |  |  |
| Hypertension | 50 (31.8) | 32 (27.4) | 18 (45) | 0.039 | 1.473 (0.73,2.976) | 0.28 |
| Gastroesophageal reflux disease | 49 (31.2) | 33 (28.2) | 16 (40) | 0.165 | / | / |
| Osteoporosis | 31 (19.7) | 23 (19.7) | 8 (20) | 0.963 | / | / |
| Dyslipidemia | 28 (17.8) | 25 (21.4) | 3 (7.5) | 0.048 | 0.603 (0.169,2.156) | 0.437 |
| Atherosclerosis | 32 (20.4) | 20 (17.1) | 12 (30) | 0.080 | / | / |
| Hepatic steatosis | 23 (14.6) | 20 (17.1) | 3 (7.5) | 0.139 | / | / |
| Osteoarthritis | 6 (3.8) | 4 (3.4) | 2 (5) | 0.645 | / | / |
| Hypothyroidism | 6 (3.8) | 4 (3.4) | 2 (5) | 0.645 | / | / |
| Type 2 diabetes mellitus | 28 (17.8) | 18 (15.4) | 10 (25) | 0.170 | / | / |
| Pulmonary infection | 20 (12.7) | 8 (6.8) | 12 (30) | ＜0.001 | 4.895 (1.834,13.062) | 0.002 |
| Ischemic stroke | 16 (10.2) | 8 (6.8) | 8 (20) | 0.030 | 2.515 (1.057,5.987) | 0.037 |
| Coronary artery disease | 22 (14) | 13 (11.1) | 9 (22.5) | 0.073 | / | / |
| Liver cyst | 10 (6.4) | 7 (6) | 3 (7.5) | 0.716 | / | / |
| Cholelithiasis | 13 (8.3) | 11 (9.4) | 2 (5) | 0.518 | / | / |
| Primary biliary cholangitis | 0 | 0 | 0 | / | / | / |
| Hyperuricemia | 13 (8.3) | 8 (6.8) | 5 (12.5) | 0.319 | / | / |
| Respiratory failure | 13 (8.3) | 10 (8.5) | 3 (7.5) | 1.000 | / | / |
| Heart failure | 6 (3.8) | 2 (1.7) | 4 (10) | 0.037 | 1.867 (0.499,6.982) | 0.354 |
| Chronic obstructive pulmonary disease | 10 (6.4) | 8 (6.8) | 2 (5) | 1.000 | / | / |
| Cancer | 5 (3.2) | 3 (2.6) | 2 (5) | 0.602 | / | / |
| **Laboratory features** |  |  |  |  |  |  |
| Leukopenia | 13 (8.3) | 10 (8.5) | 3 (7.5) | 1.000 | / | / |
| Neutropenia | 11 (7) | 9 (7.7) | 2 (5) | 0.731 | / | / |
| Lymphocytopenia | 35 (22.3) | 25 (21.4) | 10 (25) | 0.634 | / | / |
| Thrombocytopenia | 15 (9.6) | 10 (8.5) | 5 (12.5) | 0.534 | / | / |
| Anemia | 110 (70.1) | 88 (75.2) | 22 (55) | 0.016 | 1.24 (0.528,2.908) | 0.621 |
| Elevated ALT | 18 (11.5) | 15 (12.8) | 3 (7.5) | 0.566 | / | / |
| Elevated AST | 12 (7.6) | 10 (8.5) | 2 (5) | 0.732 | / | / |
| Elevated TBIL | 6 (3.8) | 5 (4.3) | 1 (2.5) | 1.000 | / | / |
| Elevated GGT | 23 (14.6) | 17 (14.5) | 6 (15) | 0.946 | / | / |
| Elevated Creatinine | 12 (7.6) | 3 (2.6) | 9 (22.5) | ＜0.001 | 2.677 (0.864,8.291) | 0.088 |
| Hypoalbuminemia | 31 (19.7) | 19 (16.2) | 12 (30) | 0.059 | / | / |
| Hyponatremia | 21 (13.4) | 13 (11.1) | 8 (20) | 0.154 | / | / |
| Hypokalemia | 6 (3.8) | 4 (3.4) | 2 (5) | 0.645 | / | / |
| Hypochloremia | 5 (3.2) | 4 (3.4) | 1 (2.5) | 1.000 | / | / |
| Hypergammaglobulinemia | 72 (45.9) | 55 (47) | 17 (42.5) | 0.621 | / | / |
| Hyper-IgG (> 16.2 g/L) | 53 (33.8) | 39 (33.3) | 14 (35) | 0.847 | / | / |
| Hyper-IgA (> 3.78 g/L) | 36 (22.9) | 28 (23.9) | 8 (20) | 0.61 | / | / |
| Hyper-IgM (> 2.63 g/L) | 7 (4.5) | 5 (4.3) | 2 (5) | 1.000 | / | / |
| Hypocomplementemia | 44 (28) | 34 (29.1) | 10 (25) | 0.622 | / | / |
| Low complement C3 (＜0.70 g/L) | 24 (15.3) | 19 (16.2) | 5 (12.5) | 0.570 | / | / |
| Low complement C4 (＜0.16 g/L) | 35 (22.3) | 26 (22.2) | 9 (22.5) | 0.971 | / | / |
| Elevated CRP (＞ 8 mg/L) | 61 (38.9) | 38 (32.5) | 23 (57.5) | 0.005 | 1.877 (0.891,3.953) | 0.098 |
| Elevated ESR (＞ 20 mm/h) | 68 (43.3) | 48 (41) | 20 (50) | 0.323 | / | / |
| ANA titres ≥1:160 | 66 (42) | 47 (40.2) | 19 (47.5) | 0.418 | / | / |
| Positive RF | 57 (36.3) | 45 (38.5) | 12 (30) | 0.337 | / | / |
| Positive anti-SSA | 69 (43.9) | 55 (47) | 14 (35) | 0.187 | / | / |
| Positive anti-Ro-52 | 57 (36.3) | 41 (35) | 16 (40) | 0.574 | / | / |
| Positive anti-SSB | 33 (21) | 26 (22.2) | 7 (17.5) | 0.527 | / | / |
| Positive anti-RNP | 6 (3.8) | 5 (4.3) | 1 (2.5) | 1.000 | / | / |
| Positive anti-CENP-B | 5 (3.2) | 2 (1.7) | 3 (7.5) | 0.105 | / | / |
| Positive anti-AMA-M2 | 7 (4.5) | 5 (4.3) | 2 (5) | 1.000 | / | / |

Data are presented as median (IQR) or n (%). Comparative analyses were conducted between with and without cancer groups of patients with primary Sjögren’s disease.

* Calculated using the *χ²* test or Fisher’s exact test for categorical variables, and the Mann-Whitney U test for continuous variables.

† Variables with *P* < 0.05 in the univariate analysis were included in the multivariable Cox regression model.

ILD: interstitial lung disease. ESSDAI score: European Alliance of Associations for Rheumatology (EULAR) Sjögren’s Syndrome Disease Activity Index score. ALT: alanine aminotransferase. AST: aspartate aminotransferase. TBIL: total bilirubin. GGT: γ-glutamyl transferase. Hyper-IgG: Hypergammaglobulinemia with elevated immunoglobulin G. Hyper-IgA: Hypergammaglobulinemia with elevated immunoglobulin A. Hyper-IgM: Hypergammaglobulinemia with elevated immunoglobulin M. Elevated CRP: elevated C-reactive protein. Elevated ESR: elevated erythrocyte sedimentation rate. HR: hazard ratio. CI: confidence intervals.

| **Supplementary Table S7. Sex-stratified penalized Cox regression with Firth’s correction for all-cause death in patients with pSD** | | | | | | |
| --- | --- | --- | --- | --- | --- | --- |
| **Subgroups** | **Variables** | **All** | **Survival** | **Death** | **Firth Cox analysis** | |
|  |  |  |  |  | **HR (95% CI)** | ***P* value**† |
| **Male** |  |  |  |  |  |  |
|  | Age (years) | 64.0 (54.0, 70.0) | 61.0 (50.0, 68.0) | 68.0 (64.0, 74.0) | 1.062 (1.017, 1.110) | 0.003 |
|  | ILD | 90 (57.3) | 56 (47.9) | 34 (85.0) | 6.535 (2.555, 16.714) | ＜0.001 |
|  | Pulmonary infection | 20 (12.7) | 8 (6.8) | 12 (30.0) | 4.648 (2.064, 10.470) | ＜0.001 |
|  | Ischemic stroke | 16 (10.2) | 8 (6.8) | 8 (20.0) | 2.807 (1.271, 6.196) | 0.018 |
| **Female** |  |  |  |  |  |  |
|  | Age (years) | 58 (49.0, 66.0) | 57 (48, 65) | 69 (61.5, 77.5) | 1.085 (1.064, 1.107) | ＜0.001 |
|  | Low C3 | 237(23.1) | 210(22.3) | 27(31.8) | 2.097 (1.268, 3.467) | 0.006 |
|  | Elevated CRP (＞8 mg/L) | 235(22.9) | 189(20.1) | 46(54.1) | 3.625 (2.317, 5.670) | ＜0.001 |
|  | Elevated TBIL | 39(3.8) | 28(3) | 11(12.9) | 4.544 (2.290, 9.020) | ＜0.001 |

Data are presented as the median (IQR) or n (%). Comparative analyses were conducted between the survival and death groups of patients with primary Sjögren’s disease. The parsimonious multivariable models were prespecified based on clinical relevance. For male patients, EPV = 10.0 (40 events/4 parameters). For female patients, EPV = 21.25 (85 events/4 parameters). Penalized Cox regression with Firth’s correction was performed as a sensitivity analysis to mitigate potential small-sample bias.

† Calculated via the Penalized Cox regression with Firth’s correction.

ILD: interstitial lung disease. C3: component 3; CRP: C-reactive protein. TBIL: total bilirubin. HR: hazard ratio. CI: confidence interval.

| **Supplementary Table S8. Univariate and multivariate Cox regression analyses of factors associated with death in female patients with pSD** | | | | | | |
| --- | --- | --- | --- | --- | --- | --- |
| **Variables** | **All (n=1025)** | **Survival (n=940)** | **Death (n=85)** | **Univariate analysis** | **Multivariate analysis** | |
|  |  |  |  | **P value*** | **HR (95%CI)** | **P value†** |
| **Clinical characteristics** |  |  |  |  |  |  |
| Age (years) | 58 (49,66) | 57 (48,65) | 69 (61.5,77.5) | ＜0.001 | 1.077 (1.051,1.105) | ＜0.001 |
| Disease duration (months) | 48 (12,108) | 48 (12,108) | 36 (5.5,120) | 0.589 | / | / |
| **Clinical manifestations** |  |  |  |  |  |  |
| Xerostomia | 883 (86.1) | 810 (86.2) | 73 (85.9) | 0.941 | / | / |
| Xerophthalmia | 815 (79.5) | 748 (79.6) | 67 (78.8) | 0.870 | / | / |
| Fatigue | 505 (49.3) | 458 (48.7) | 47 (55.3) | 0.246 | / | / |
| Fever | 112 (10.9) | 94 (10) | 18 (21.2) | 0.002 | 1.333 (0.694,2.56) | 0.388 |
| Purpuric rash | 80 (7.8) | 72 (7.7) | 8 (9.4) | 0.564 | / | / |
| Arthralgia | 365 (35.6) | 344 (36.6) | 21 (24.7) | 0.028 | 0.582 (0.332,1.019) | 0.058 |
| Arthritis | 93 (9.1) | 90 (9.6) | 3 (3.5) | 0.063 | / | / |
| Dyspnea | 262 (25.6) | 221 (23.5) | 41 (48.2) | ＜0.001 | 1.741 (0.958,3.164) | 0.069 |
| Cough | 308 (30) | 266 (28.3) | 42 (49.4) | ＜0.001 | 1.485 (0.822,2.682) | 0.190 |
| Lymphadenopathy | 185 (18) | 169 (18) | 16 (18.8) | 0.846 | / | / |
| Parotid enlargement | 76 (7.4) | 72 (7.7) | 4 (4.7) | 0.320 | / | / |
| Splenomegaly | 48 (4.7) | 40 (4.3) | 8 (9.4) | 0.053 | 1.251 (0.479,3.268) | 0.648 |
| Hemorrhage | 66 (6.4) | 56 (6) | 10 (11.8) | 0.037 | 2.089 (0.903,4.834) | 0.085 |
| ILD | 282 (27.5) | 243 (25.9) | 39 (45.9) | ＜0.001 | 1.364 (0.784,2.37) | 0.272 |
| ESSDAI score | 6 (3,13) | 6 (3,12) | 13 (6,21) | ＜0.001 | 1.027 (0.991,1.065) | 0.145 |
| **Comorbidities** |  |  |  |  |  |  |
| Hypertension | 252 (24.6) | 221 (23.5) | 31 (36.5) | 0.008 | 1.633 (0.929,2.87) | 0.088 |
| Gastroesophageal reflux disease | 235 (22.9) | 216 (23) | 19 (22.4) | 0.895 | / | / |
| Osteoporosis | 242 (23.6) | 213 (22.7) | 29 (34.1) | 0.017 | 1.273 (0.756,2.142) | 0.364 |
| Dyslipidemia | 189 (18.4) | 172 (18.3) | 17 (20) | 0.698 | / | / |
| Atherosclerosis | 135 (13.2) | 120 (12.8) | 15 (17.6) | 0.203 | / | / |
| Hepatic steatosis | 132 (12.9) | 128 (13.6) | 4 (4.7) | 0.019 | 0.442 (0.157,1.243) | 0.122 |
| Osteoarthritis | 131 (12.8) | 124 (13.2) | 7 (8.2) | 0.190 | / | / |
| Hypothyroidism | 120 (11.7) | 105 (11.2) | 15 (17.6) | 0.075 | / | / |
| Type 2 diabetes mellitus | 80 (7.8) | 67 (7.1) | 13 (15.3) | 0.007 | 1.234 (0.624,2.442) | 0.546 |
| Pulmonary infection | 84 (8.2) | 67 (7.1) | 17 (20) | ＜0.001 | 1.149 (0.595,2.221) | 0.679 |
| Ischemic stroke | 88 (8.6) | 73 (7.8) | 15 (17.6) | 0.002 | 1.075 (0.561,2.062) | 0.827 |
| Coronary artery disease | 75 (7.3) | 61 (6.5) | 14 (16.5) | 0.001 | 1.174 (0.528,2.61) | 0.694 |
| Liver cyst | 74 (7.2) | 70 (7.4) | 4 (4.7) | 0.350 | / | / |
| Cholelithiasis | 54 (5.3) | 50 (5.3) | 4 (4.7) | 1.000 | / | / |
| Primary biliary cholangitis | 61 (6) | 53 (5.6) | 8 (9.4) | 0.159 | / | / |
| Hyperuricemia | 32 (3.1) | 26 (2.8) | 6 (7.1) | 0.043 | 1.006 (0.331,3.056) | 0.991 |
| Respiratory failure | 29 (2.8) | 17 (1.8) | 12 (14.1) | ＜0.001 | 1.695 (0.663,4.333) | 0.270 |
| Heart failure | 35 (3.4) | 23 (2.4) | 12 (14.1) | ＜0.001 | 1.75 (0.776,3.948) | 0.177 |
| Chronic obstructive pulmonary disease | 26 (2.5) | 19 (2) | 7 (8.2) | 0.004 | 1.004 (0.379,2.655) | 0.994 |
| Cancer | 18 (1.8) | 14 (1.5) | 4 (4.7) | 0.031 | 1.017 (0.297,3.489) | 0.978 |
| **Laboratory features** |  |  |  |  |  |  |
| Leukopenia | 207 (20.2) | 193 (20.5) | 14 (16.5) | 0.372 | / | / |
| Neutropenia | 183 (17.9) | 175 (18.6) | 8 (9.4) | 0.034 | 0.577 (0.245,1.362) | 0.210 |
| Lymphocytopenia | 265 (25.9) | 232 (24.7) | 33 (38.8) | 0.004 | 1.164 (0.654,2.071) | 0.606 |
| Thrombocytopenia | 159 (15.5) | 136 (14.5) | 23 (27.1) | 0.002 | 1.099 (0.547,2.208) | 0.791 |
| Anemia | 316 (30.8) | 271 (28.8) | 45 (52.9) | ＜0.001 | 1.196 (0.687,2.08) | 0.527 |
| Elevated ALT | 117 (11.4) | 105 (11.2) | 12 (14.1) | 0.413 | / | / |
| Elevated AST | 172 (16.8) | 153 (16.3) | 19 (22.4) | 0.151 | / | / |
| Elevated TBIL | 39 (3.8) | 28 (3) | 11 (12.9) | ＜0.001 | 4.255 (1.675,10.807) | 0.002 |
| Elevated GGT | 185 (18) | 157 (16.7) | 28 (32.9) | ＜0.001 | 1.331 (0.774,2.289) | 0.300 |
| Elevated Creatinine | 58 (5.7) | 48 (5.1) | 10 (11.8) | 0.023 | 1.081 (0.473,2.471) | 0.853 |
| Hypoalbuminemia | 105 (10.2) | 77 (8.2) | 28 (32.9) | ＜0.001 | 1.33 (0.698,2.536) | 0.386 |
| Hyponatremia | 116 (11.3) | 88 (9.4) | 28 (32.9) | ＜0.001 | 1.828 (0.965,3.464) | 0.064 |
| Hypokalemia | 97 (9.5) | 89 (9.5) | 8 (9.4) | 0.986 | / | / |
| Hypochloremia | 25 (2.4) | 14 (1.5) | 11 (12.9) | ＜0.001 | 2.117 (0.907,4.946) | 0.083 |
| Hypergammaglobulinemia | 604 (58.9) | 556 (59.1) | 48 (56.5) | 0.631 | / | / |
| Hyper-IgG (> 16.2 g/L) | 493 (48.1) | 453 (48.2) | 40 (47.1) | 0.841 | / | / |
| Hyper-IgA (> 3.78 g/L) | 270 (26.3) | 241 (25.6) | 29 (34.1) | 0.089 | / | / |
| Hyper-IgM (> 2.63 g/L) | 81 (7.9) | 72 (7.7) | 9 (10.6) | 0.338 | / | / |
| Hypocomplementemia | 453 (44.2) | 414 (44) | 39 (45.9) | 0.744 | / | / |
| Low complement C3 (＜0.70 g/L) | 237 (23.1) | 210 (22.3) | 27 (31.8) | 0.048 | 2.057 (1.157,3.657) | 0.014 |
| Low complement C4 (＜0.16 g/L) | 374 (36.5) | 343 (36.5) | 31 (36.5) | 0.997 | / | / |
| Elevated CRP (＞ 8 mg/L) | 235 (22.9) | 189 (20.1) | 46 (54.1) | ＜0.001 | 2.491 (1.391,4.461) | 0.002 |
| Elevated ESR (＞ 20 mm/h) | 534 (52.1) | 476 (50.6) | 58 (68.2) | 0.002 | 1.348 (0.727,2.499) | 0.343 |
| ANA titres ≥1:160 | 667 (65.1) | 607 (64.6) | 60 (70.6) | 0.265 | / | / |
| Positive RF | 485 (47.3) | 444 (47.2) | 41 (48.2) | 0.859 | / | / |
| Positive anti-SSA | 774 (75.5) | 711 (75.6) | 63 (74.1) | 0.755 | / | / |
| Positive anti-Ro-52 | 622 (60.7) | 573 (61) | 49 (57.6) | 0.550 | / | / |
| Positive anti-SSB | 299 (29.2) | 277 (29.5) | 22 (25.9) | 0.486 | / | / |
| Positive anti-RNP | 78 (7.6) | 75 (8) | 3 (3.5) | 0.138 | / | / |
| Positive anti-CENP-B | 98 (9.6) | 87 (9.3) | 11 (12.9) | 0.268 | / | / |
| Positive anti-AMA-M2 | 96 (9.4) | 91 (9.7) | 5 (5.9) | 0.250 | / | / |

Data are presented as median (IQR) or n (%). Comparative analyses were conducted between with and without cancer groups of patients with primary Sjögren’s disease.

* Calculated using the *χ²* test or Fisher’s exact test for categorical variables, and the Mann-Whitney U test for continuous variables.

† Variables with *P* < 0.05 in the univariate analysis were included in the multivariable Cox regression model.

ILD: interstitial lung disease. ESSDAI score: European Alliance of Associations for Rheumatology (EULAR) Sjögren’s Syndrome Disease Activity Index score. ALT: alanine aminotransferase. AST: aspartate aminotransferase. TBIL: total bilirubin. GGT: γ-glutamyl transferase. Hyper-IgG: Hypergammaglobulinemia with elevated immunoglobulin G. Hyper-IgA: Hypergammaglobulinemia with elevated immunoglobulin A. Hyper-IgM: Hypergammaglobulinemia with elevated immunoglobulin M. Elevated CRP: elevated C-reactive protein. Elevated ESR: elevated erythrocyte sedimentation rate. HR: hazard ratio. CI: confidence intervals.

| **Supplementary Table S9. Univariate and multivariate Cox regression analyses of factors associated with cancer in male patients with pSD** | | | | | | |
| --- | --- | --- | --- | --- | --- | --- |
| **Variables** | **All (n=152)** | **Without cancer (n=142)** | **With cancer (n=10)** | **Univariate analysis** | **Multivariate analysis** |  |
|  |  |  |  | ***P* value*** | **HR (95%CI)** | ***P* value**† |
| **Clinical characteristics** |  |  |  |  |  |  |
| Age (years) | 64 (54,70) | 63.5 (53.8,69.0) | 70 (53.8,73.3) | 0.181 | / | / |
| Disease duration (months) | 12 (3,48) | 12.0 (3.0,48.0) | 24.0 (1.0,27.0) | 0.751 | / | / |
| **Clinical manifestations** |  |  |  |  |  |  |
| Xerostomia | 107 (70.4) | 98 (69.0) | 9 (90.0) | 0.282 | / | / |
| Xerophthalmia | 100 (65.8) | 93 (65.5) | 7 (70.0) | 1.000 | / | / |
| Fatigue | 57 (37.5) | 54 (38.0) | 3 (30.0) | 0.744 | / | / |
| Fever | 24 (15.8) | 21 (14.8) | 3 (30.0) | 0.195 | / | / |
| Purpuric rash | 7 (4.6) | 6 (4.2) | 1 (10.0) | 0.385 | / | / |
| Arthralgia | 28 (18.4) | 28 (19.7) | 0 (0.0) | 0.209 | / | / |
| Arthritis | 8 (5.3) | 8 (5.6) | 0 (0.0) | 1.000 | / | / |
| Dyspnea | 71 (46.7) | 67 (47.2) | 4 (40.0) | 0.751 | / | / |
| Cough | 83 (54.6) | 79 (55.6) | 4 (40.0) | 0.513 | / | / |
| Lymphadenopathy | 25 (16.4) | 24 (16.9) | 1 (10.0) | 1.000 | / | / |
| Parotid enlargement | 11 (7.2) | 10 (7.0) | 1 (10.0) | 0.539 | / | / |
| Splenomegaly | 5 (3.3) | 4 (2.8) | 1 (10.0) | 0.292 | / | / |
| Hemorrhage | 8 (5.3) | 8 (5.6) | 0 (0.0) | 1.000 | / | / |
| ILD | 86 (56.6) | 79 (55.6) | 7 (70.0) | 0.515 | / | / |
| ESSDAI score | 10.0 (3.0,16.8) | 10.0 (3.0,16.0) | 9 (4,18.5) | 0.726 | / | / |
| **Comorbidities** |  |  |  |  | / | / |
| Hypertension | 48 (31.6) | 44 (31.0) | 4 (40.0) | 0.726 | / | / |
| Gastroesophageal reflux disease | 47 (30.9) | 44 (31.0) | 3 (30.0) | 1.000 | / | / |
| Osteoporosis | 30 (19.7) | 29 (20.4) | 1 (10.0) | 0.688 | / | / |
| Dyslipidemia | 27 (17.8) | 27 (19.0) | 0 (0.0) | 0.21 | / | / |
| Atherosclerosis | 30 (19.7) | 29 (20.4) | 1 (10.0) | 0.688 | / | / |
| Hepatic steatosis | 23 (15.1) | 22 (15.5) | 1 (10.0) | 1.000 | / | / |
| Osteoarthritis | 5 (3.3) | 5 (3.5) | 0 (0.0) | 1.000 | / | / |
| Hypothyroidism | 5 (3.3) | 5 (3.5) | 0 (0.0) | 1.000 | / | / |
| Type 2 diabetes mellitus | 28 (18.4) | 25 (17.6) | 3 (30.0) | 0.393 | / | / |
| Pulmonary infection | 19 (12.5) | 16 (11.3) | 3 (30.0) | 0.113 | / | / |
| Ischemic stroke | 15 (9.9) | 15 (10.6) | 0 (0) | 0.599 | / | / |
| Coronary artery disease | 20 (13.2) | 20 (14.1) | 0 (0) | 0.361 | / | / |
| Liver cyst | 10 (6.6) | 8 (5.6) | 2 (20) | 0.132 | / | / |
| Cholelithiasis | 13 (8.6) | 12 (8.5) | 1 (10) | 1.000 | / | / |
| Primary biliary cholangitis | 0 (0.0) | 0 (0.0) | 0 (0.0) | / | / | / |
| Hyperuricemia | 13 (8.6) | 13 (9.2) | 0 (0.0) | 1.000 | / | / |
| Respiratory failure | 13 (8.6) | 12 (8.5) | 1 (10.0) | 1.000 | / | / |
| Heart failure | 5 (3.3) | 5 (3.5) | 0 (0.0) | 1.000 | / | / |
| Chronic obstructive pulmonary disease | 10 (6.6) | 9 (6.3) | 1 (10.0) | 0.505 | / | / |
| Cancer | 0 (0.0) | 0 (0.0) | 0 (0.0) | / | / | / |
| **Laboratory features** |  |  |  |  | / | / |
| Leukopenia | 13 (8.6) | 13 (9.2) | 0 (0.0) | 1.000 | / | / |
| Neutropenia | 11 (7.2) | 11 (7.7) | 0 (0.0) | 1.000 | / | / |
| Lymphocytopenia | 35 (23) | 32 (22.5) | 3 (30) | 0.697 | / | / |
| Thrombocytopenia | 14 (9.2) | 14 (9.9) | 0 (0.0) | 0.600 | / | / |
| Anemia | 106 (69.7) | 98 (69) | 8 (80) | 0.724 | / | / |
| Elevated ALT | 18 (11.8) | 16 (11.3) | 2 (20) | 0.336 | / | / |
| Elevated AST | 12 (7.9) | 12 (8.5) | 0 (0.0) | 1.000 | / | / |
| Elevated TBIL | 5 (3.3) | 5 (3.5) | 0 (0.0) | 1.000 | / | / |
| Elevated GGT | 22 (14.5) | 18 (12.7) | 4 (40) | 0.039 | 3.307 (0.932,11.729) | 0.064 |
| Elevated Creatinine | 11 (7.2) | 11 (7.7) | 0 (0.0) | 1.000 | / | / |
| Hypoalbuminemia | 30 (19.7) | 27 (19.0) | 3 (30) | 0.415 | / | / |
| Hyponatremia | 20 (13.2) | 18 (12.7) | 2 (20) | 0.622 | / | / |
| Hypokalemia | 6 (3.9) | 6 (4.2) | 0 (0.0) | 1.000 | / | / |
| Hypochloremia | 4 (2.6) | 3 (2.1) | 1 (10.0) | 0.240 | / | / |
| Hypergammaglobulinemia | 72 (47.4) | 66 (46.5) | 6 (60.0) | 0.519 | / | / |
| Hyper-IgG (> 16.2 g/L) | 53 (34.9) | 49 (34.5) | 4 (40.0) | 0.740 | / | / |
| Hyper-IgA (> 3.78 g/L) | 36 (23.7) | 33 (23.2) | 3 (30.0) | 0.701 | / | / |
| Hyper-IgM (> 2.63 g/L) | 7 (4.6) | 7 (4.9) | 0 (0.0) | 1.000 | / | / |
| Hypocomplementemia | 43 (28.3) | 39 (27.5) | 4 (40.0) | 0.470 | / | / |
| Low complement C3 (＜0.70 g/L) | 23 (15.1) | 23 (16.2) | 0 (0.0) | 0.361 | / | / |
| Low complement C4 (＜0.16 g/L) | 35 (23.0) | 31 (21.8) | 4 (40.0) | 0.240 | / | / |
| Elevated CRP (＞ 8 mg/L) | 59 (38.8) | 54 (38.0) | 5 (50.0) | 0.512 | / | / |
| Elevated ESR (＞ 20 mm/h) | 68 (44.7) | 63 (44.4) | 5 (50.0) | 0.753 | / | / |
| ANA titres ≥1:160 | 65 (42.8) | 59 (41.5) | 6 (60.0) | 0.327 | / | / |
| Positive RF | 56 (36.8) | 54 (38.0) | 2 (20.0) | 0.325 | / | / |
| Positive anti-SSA | 66 (43.4) | 62 (43.7) | 4 (40.0) | 1.000 | / | / |
| Positive anti-Ro-52 | 54 (35.5) | 50 (35.2) | 4 (40.0) | 0.744 | / | / |
| Positive anti-SSB | 33 (21.7) | 31 (21.8) | 2 (20.0) | 1.000 | / | / |
| Positive anti-RNP | 6 (3.9) | 5 (3.5) | 1 (10.0) | 0.340 | / | / |
| Positive anti-CENP-B | 4 (2.6) | 3 (2.1) | 1 (10.0) | 0.240 | / | / |
| Positive anti-AMA-M2 | 7 (4.6) | 5 (3.5) | 2 (20.0) | 0.069 | / | / |

Data are presented as median (IQR) or n (%). Comparative analyses were conducted between with and without cancer groups of patients with primary Sjögren’s disease.

* Calculated using the *χ²* test or Fisher’s exact test for categorical variables, and the Mann-Whitney U test for continuous variables.

† Variables with *P* < 0.05 in the univariate analysis were included in the multivariable Cox regression model.

ILD: interstitial lung disease. ESSDAI score: European Alliance of Associations for Rheumatology (EULAR) Sjögren’s Syndrome Disease Activity Index score. ALT: alanine aminotransferase. AST: aspartate aminotransferase. TBIL: total bilirubin. GGT: γ-glutamyl transferase. Hyper-IgG: Hypergammaglobulinemia with elevated immunoglobulin G. Hyper-IgA: Hypergammaglobulinemia with elevated immunoglobulin A. Hyper-IgM: Hypergammaglobulinemia with elevated immunoglobulin M. Elevated CRP: elevated C-reactive protein. Elevated ESR: elevated erythrocyte sedimentation rate. HR: hazard ratio. CI: confidence intervals.

| **Supplementary Table S10. Sex-stratified penalized Cox regression with Firth’s correction for cancer in patients with pSD** | | | | | | |
| --- | --- | --- | --- | --- | --- | --- |
| **Subgroups** | **Variables** | **All** | **Without cancer** | **With cancer** | **Firth Cox analysis** | |
|  |  |  |  |  | **HR (95% CI)** | ***P* value**† |
| **Male** |  |  |  |  |  |  |
|  | Age (years) | 64.0 (54.0, 70.0) | 63.5 (53.75, 69.0) | 70.0 (53.8, 73.3) | 1.029 (0.976, 1.085) | 0.260 |
|  | Disease duration (months) | 12.0 (3.0, 48.0) | 12.0 (3.0, 48.0) | 24.0 (1.0, 27.0) | 1.005 (0.936, 1.080) | 0.880 |
| **Female** |  |  |  |  |  |  |
|  | Disease duration (months) | 48 (12,108) | 48 (12,108) | 96 (36,156) | 1.006 (1.002, 1.009) | 0.002 |
|  | Lymphadenopathy | 182 (18.1) | 172 (17.5) | 10 (43.5) | 4.593 (2.032, 10.383) | ＜0.001 |
|  | Lymphocytopenia | 259 (25.7) | 248 (25.2) | 11 (47.8) | 2.199 (0.998, 4.848) | 0.051 |
|  | Elevated TBIL | 37 (3.7) | 34 (3.5) | 3 (13.0) | 4.655 (1.395,15.531) | 0.029 |

Data are presented as the median (IQR) or n (%). Comparative analyses were conducted between the groups of patients with primary Sjögren’s disease with and without cancer. Due to the limited number of cancer events, only parsimonious models were considered. For male patients, EPV was 5.0 (10 events/2 parameters), and penalized Cox regression with Firth’s correction was applied as a sensitivity analysis. For female patients, EPV was 6.5 (26 events/4 parameters), and penalized Cox regression with Firth’s correction was likewise performed to mitigate potential small-sample bias.

† Calculated via the Penalized Cox regression with Firth’s correction.

TBIL: total bilirubin. HR: hazard ratio. CI: confidence interval.

| **Supplementary Table S11. Univariate and multivariate Cox regression analyses of factors associated with cancer in female patients with pSD** | | | | | | |
| --- | --- | --- | --- | --- | --- | --- |
| **Variables** | **All (n=1007)** | **Without cancer (n=984)** | **With cancer (n=23)** | **Univariate analysis** | **Multivariate analysis** |  |
|  |  |  |  | **P value*** | **HR (95% CI)** | **P value†** |
| **Clinical characteristics** |  |  |  |  |  |  |
| Age (years) | 61 (54,70) | 58 (48,66) | 61 (54,70) | 0.127 | / | / |
| Disease duration (months) | 96 (36,156) | 48 (12,108) | 96 (36,156) | 0.016 | 1.005 (1.002,1.008) | 0.002 |
| **Clinical manifestations** |  |  |  |  |  |  |
| Xerostomia | 870 (86.4) | 851 (86.5) | 19 (82.6) | 0.540 | / | / |
| Xerophthalmia | 801 (79.5) | 781 (79.4) | 20 (87) | 0.600 | / | / |
| Fatigue | 499 (49.6) | 486 (49.4) | 13 (56.5) | 0.499 | / | / |
| Fever | 109 (10.8) | 106 (10.8) | 3 (13) | 0.730 | / | / |
| Purpuric rash | 79 (7.8) | 77 (7.8) | 2 (8.7) | 0.700 | / | / |
| Arthralgia | 363 (36) | 352 (35.8) | 11 (47.8) | 0.002 | 1.285 (0.545,3.029) | 0.567 |
| Arthritis | 93 (9.2) | 88 (8.9) | 5 (21.7) | 0.053 | / | / |
| Dyspnea | 255 (25.3) | 252 (25.6) | 3 (13) | 0.002 | 0.681 (0.198,2.347) | 0.543 |
| Cough | 302 (30) | 296 (30.1) | 6 (26.1) | 0.679 | / | / |
| Lymphadenopathy | 182 (18.1) | 172 (17.5) | 10 (43.5) | 0.004 | 4.748 (1.989,11.334) | ＜0.001 |
| Parotid enlargement | 75 (7.4) | 73 (7.4) | 2 (8.7) | 0.687 | / | / |
| Splenomegaly | 46 (4.6) | 44 (4.5) | 2 (8.7) | 0.283 | / | / |
| Hemorrhage | 64 (6.4) | 60 (6.1) | 4 (17.4) | 0.053 | / | / |
| ILD | 274 (27.2) | 269 (27.3) | 5 (21.7) | 0.551 | / | / |
| ESSDAI score | 6 (3,13) | 6 (3,13) | 7 (4,12) | 0.566 | / | / |
| **Comorbidities** |  |  |  |  | / | / |
| Hypertension | 251 (24.9) | 244 (24.8) | 7 (30.4) | 0.537 | / | / |
| Gastroesophageal reflux disease | 232 (23) | 224 (22.8) | 8 (34.8) | 0.208 | / | / |
| Osteoporosis | 240 (23.8) | 232 (23.6) | 8 (34.8) | 0.212 | / | / |
| Dyslipidemia | 186 (18.5) | 184 (18.7) | 2 (8.7) | 0.286 | / | / |
| Atherosclerosis | 134 (13.3) | 130 (13.2) | 4 (17.4) | 0.533 | / | / |
| Hepatic steatosis | 132 (13.1) | 126 (12.8) | 6 (26.1) | 0.107 | / | / |
| Osteoarthritis | 131 (13) | 126 (12.8) | 5 (21.7) | 0.208 | / | / |
| Hypothyroidism | 120 (11.9) | 115 (11.7) | 5 (21.7) | 0.180 | / | / |
| Type 2 diabetes mellitus | 79 (7.8) | 77 (7.8) | 2 (8.7) | 0.700 | / | / |
| Pulmonary infection | 84 (8.3) | 81 (8.2) | 3 (13) | 0.431 | / | / |
| Ischemic stroke | 86 (8.5) | 82 (8.3) | 4 (17.4) | 0.128 | / | / |
| Coronary artery disease | 75 (7.4) | 73 (7.4) | 2 (8.7) | 0.687 | / | / |
| Liver cyst | 72 (7.1) | 68 (6.9) | 4 (17.4) | 0.075 | / | / |
| Cholelithiasis | 51 (5.1) | 48 (4.9) | 3 (13) | 0.106 | / | / |
| Primary biliary cholangitis | 60 (6) | 56 (5.7) | 4 (17.4) | 0.043 | 2.635 (0.853,8.145) | 0.092 |
| Hyperuricemia | 32 (3.2) | 32 (3.3) | 0 (0) | 1.000 | / | / |
| Respiratory failure | 29 (2.9) | 29 (2.9) | 0 (0) | 1.000 | / | / |
| Heart failure | 34 (3.4) | 33 (3.4) | 1 (4.3) | 0.550 | / | / |
| Chronic obstructive pulmonary disease | 26 (2.6) | 26 (2.6) | 0 (0) | 1.000 | / | / |
| **Laboratory features** |  |  |  |  |  |  |
| Leukopenia | 201 (20) | 194 (19.7) | 7 (30.4) | 0.195 | / | / |
| Neutropenia | 179 (17.8) | 174 (17.7) | 5 (21.7) | 0.584 | / | / |
| Lymphocytopenia | 11 (47.8) (0) | 259 (25.7) (0) | 248 (25.2) (0) | 0.014 | 2.597 (1.091,6.18) | 0.031 |
| Thrombocytopenia | 153 (15.2) | 148 (15) | 5 (21.7) | 0.376 | / | / |
| Anemia | 308 (30.6) | 298 (30.3) | 10 (43.5) | 0.175 | / | / |
| Elevated ALT | 113 (11.2) | 111 (11.3) | 2 (8.7) | 1.000 | / | / |
| Elevated AST | 166 (16.5) | 160 (16.3) | 6 (26.1) | 0.249 | / | / |
| Elevated TBIL | 37 (3.7) | 34 (3.5) | 3 (13) | 0.049 | 4.27 (1.09,16.726) | 0.037 |
| Elevated GGT | 181 (18) | 174 (17.7) | 7 (30.4) | 0.163 | / | / |
| Elevated Creatinine | 56 (5.6) | 56 (5.7) | 0 (0) | 0.634 | / | / |
| Hypoalbuminemia | 102 (10.1) | 101 (10.3) | 1 (4.3) | 0.722 | / | / |
| Hyponatremia | 114 (11.3) | 110 (11.2) | 4 (17.4) | 0.319 | / | / |
| Hypokalemia | 96 (9.5) | 95 (9.7) | 1 (4.3) | 0.717 | / | / |
| Hypochloremia | 25 (2.5) | 22 (2.2) | 3 (13) | 0.017 | 3.684 (1.026,13.231) | 0.046 |
| Hypergammaglobulinemia | 596 (59.2) | 584 (59.3) | 12 (52.2) | 0.489 | / | / |
| Hyper-IgG (> 16.2 g/L) | 487 (48.4) | 477 (48.5) | 10 (43.5) | 0.635 | / | / |
| Hyper-IgA (> 3.78 g/L) | 267 (26.5) | 258 (26.2) | 9 (39.1) | 0.166 | / | / |
| Hyper-IgM (> 2.63 g/L) | 79 (7.8) | 76 (7.7) | 3 (13) | 0.417 | / | / |
| Hypocomplementemia | 446 (44.3) | 437 (44.4) | 9 (39.1) | 0.614 | / | / |
| Low complement C3 (＜0.70 g/L) | 231 (22.9) | 226 (23) | 5 (21.7) | 0.890 | / | / |
| Low complement C4 (＜0.16 g/L) | 369 (36.6) | 361 (36.7) | 8 (34.8) | 0.009 | 0.643 (0.261,1.585) | 0.338 |
| Elevated CRP (＞ 8 mg/L) | 225 (22.3) | 219 (22.3) | 6 (26.1) | 0.663 | / | / |
| Elevated ESR (＞ 20 mm/h) | 521 (51.7) | 509 (51.7) | 12 (52.2) | 0.966 | / | / |
| ANA titres ≥1:160 | 656 (65.1) | 640 (65) | 16 (69.6) | 0.653 | / | / |
| Positive RF | 478 (47.5) | 467 (47.5) | 11 (47.8) | 0.972 | / | / |
| Positive anti-SSA | 761 (75.6) | 743 (75.5) | 18 (78.3) | 0.761 | / | / |
| Positive anti-Ro-52 | 611 (60.7) | 595 (60.5) | 16 (69.6) | 0.377 | / | / |
| Positive anti-SSB | 294 (29.2) | 285 (29) | 9 (39.1) | 0.289 | / | / |
| Positive anti-RNP | 76 (7.5) | 72 (7.3) | 4 (17.4) | 0.089 | / | / |
| Positive anti-CENP-B | 96 (9.5) | 91 (9.2) | 5 (21.7) | 0.060 | / | / |
| Positive anti-AMA-M2 | 95 (9.4) | 92 (9.3) | 3 (13) | 0.471 | / | / |

Data are presented as median (IQR) or n (%). Comparative analyses were conducted between with and without cancer groups of patients with primary Sjögren’s disease.

* Calculated using the *χ²* test or Fisher’s exact test for categorical variables, and the Mann-Whitney U test for continuous variables.

† Variables with *P* < 0.05 in the univariate analysis were included in the multivariable Cox regression model.

ILD: interstitial lung disease. ESSDAI score: European Alliance of Associations for Rheumatology (EULAR) Sjögren’s Syndrome Disease Activity Index score. ALT: alanine aminotransferase. AST: aspartate aminotransferase. TBIL: total bilirubin. GGT: γ-glutamyl transferase. Hyper-IgG: Hypergammaglobulinemia with elevated immunoglobulin G. Hyper-IgA: Hypergammaglobulinemia with elevated immunoglobulin A. Hyper-IgM: Hypergammaglobulinemia with elevated immunoglobulin M. Elevated CRP: elevated C-reactive protein. Elevated ESR: elevated erythrocyte sedimentation rate. HR: hazard ratio. CI: confidence intervals.
