# Supplementary material for: Sex differences in primary Sjögren’s disease: prognostic impact on mortality and cancer
Source: Biol Sex Differ. 2026 Jan 14;17:25. doi: 10.1186/s13293-026-00827-7 (PMC12888568; doi:10.1186/s13293-026-00827-7)
Supplement: Supplementary file 2 — Additional file 2 [file 13293_2026_827_MOESM2_ESM.docx]

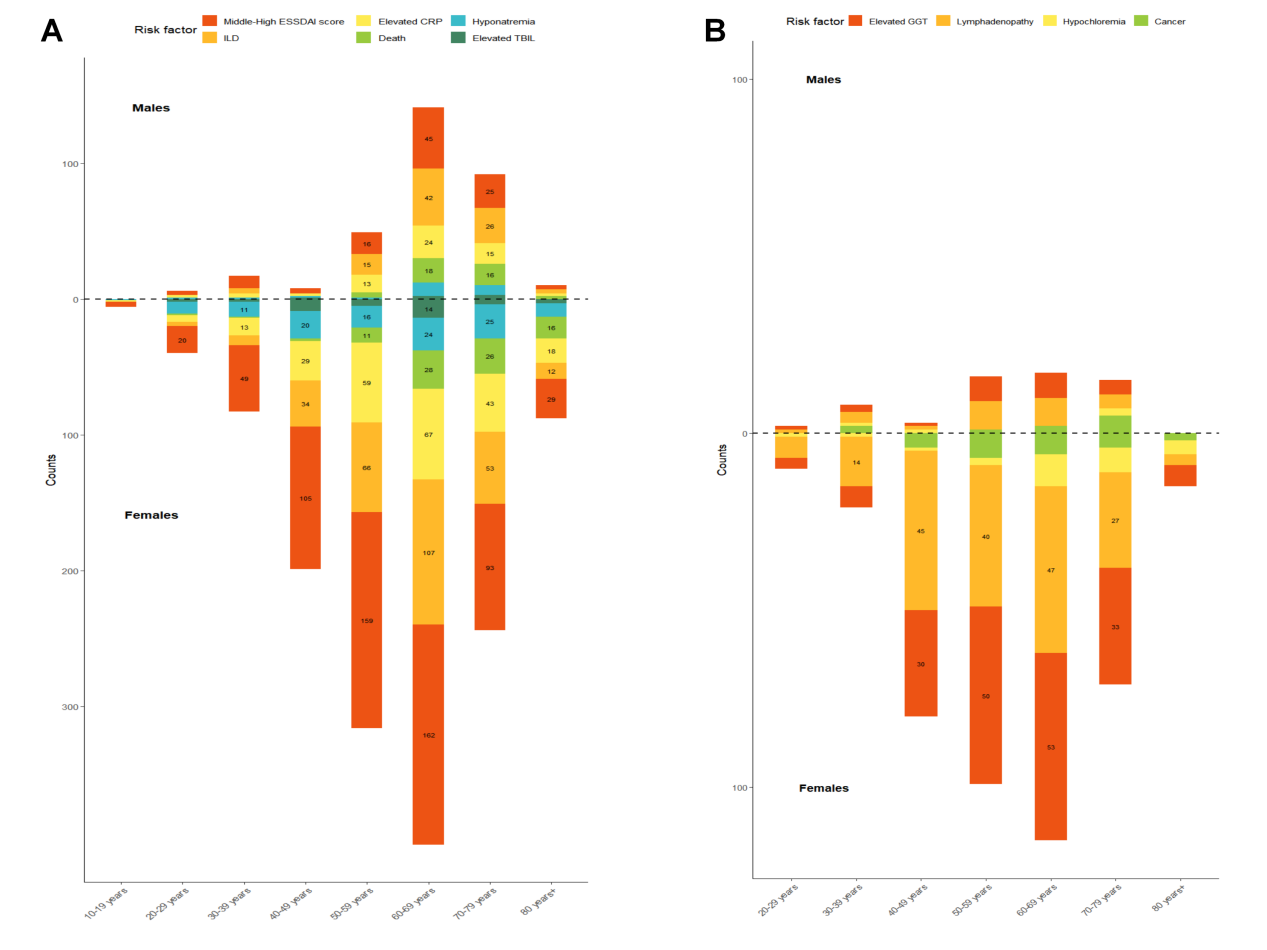


**Supplementary Figure S1.** Sex-specific prognostic factors and stepwise Cox regression for death in patients with pSD. (A) Distribution of major death-related prognostic factors stratified by age and sex. Each stacked bar represents the cumulative count of patients carrying ≥1 prognostic factor. Only counts ≥10 are annotated within each bar. (B) Distribution of major cancer-related prognostic factors stratified by age and sex. Stacked bars represent the cumulative counts of patients carrying ≥1 prognostic factor. Only counts ≥10 are annotated within each bar.

ILD: interstitial lung disease. ESSDAI: European Alliance of Associations for Rheumatology (EULAR) Sjögren’s Syndrome Disease Activity Index score. CRP: C-reactive protein. TBIL: total bilirubin. GGT: γ-glutamyl transpeptidase. HR: hazard ratio. 95% CI: 95% confidence interval.
